# Characterization of the brain lipidome associated with frontotemporal lobar degeneration MAPT P301S mutation

**DOI:** 10.1016/j.jlr.2025.100952

**Published:** 2025-11-27

**Authors:** Almudena Maroto-Juanes, Thomas Vogels, Sascha Koppes-den Hertog, Maarten Loos, Dieter Lütjohann, Martin Giera, Rik van der Kant

**Affiliations:** 1 Department of Functional Genomics, Center for Neurogenomics and Cognitive Research (CNCR), Vrije University (VU), Amsterdam, the Netherlands; 2 Alzheimer Center Amsterdam, Department of Neurology, Amsterdam University Medical Center, Amsterdam Neuroscience, Amsterdam, The Netherlands; 3 InnoSer, Leiden, the Netherlands; 4 Institute for Clinical Chemistry and Clinical Pharmacology, University Hospital Bonn, Bonn, Germany; 5 Center for Proteomics and Metabolomics, Leiden University Medical Center, Leiden, the Netherlands

**Keywords:** frontotemporal lobar degeneration, tau, lipidomics, neutral lipids, cholesterol metabolism

## Abstract

Mutations in microtubule-associated protein Tau (*MAPT*), the gene that codes for the protein Tau, cause frontotemporal lobar degeneration (FTLD) with phenotypes ranging from behavioral changes to cognitive impairment and parkinsonism. Recently, lipid changes have been heavily implicated in synucleinopathies and secondary tauopathies such as Alzheimer’s disease. Whether mutations in *MAPT* or accumulation of hyperphosphorylated Tau (pTau) can contribute to lipid changes in primary tauopathies is unknown. Here, we examine the effect of the FTLD-associated mutation *MAPT* P301S on brain lipid metabolism in a Tau transgenic mouse model. We find that the *MAPT* P301S mutation drives increased levels of diglycerides and hexosylceramides and lactosylceramides while reducing triglycerides, specifically those triglyceride species containing monounsaturated fatty acids, but does not affect cholesterol metabolism prior to pTau accumulation. Strikingly, with increasing accumulation of pTau, neutral lipids such as cholesteryl esters and triglycerides start to accumulate in the brain of mutant mice, as also reported in the Alzheimer’s disease and FTLD brain. Furthermore, with increasing buildup of pTau, we observe decreased cholesterol synthesis and turnover to 24S-hydroxycholesterol. Overall our data indicates that the *MAPT* P301S mutation and accumulation of pTau are associated with distinct brain lipidomes in vivo.

Primary tauopathies are a group of neurodegenerative disorders characterized by the presence of aggregated Tau inclusions in the brain (1). In tauopathies, Tau protein undergoes posttranslational modifications—primarily hyperphosphorylation—that make it more prone to aggregation into neurofibrillary tangles (2, 3), leading to neuronal loss and cerebral atrophy (4, 5). In primary tauopathies, *MAPT* mutations are known to cause frontotemporal lobar degeneration (FTLD) with Tau pathology, a heterogeneous term that encompasses diseases such as progressive supranuclear palsy (PSP), corticobasal degeneration, or Pick’s disease, which present clinical and pathological heterogeneity, with clinical manifestations ranging from cognitive decline to movement disorders (6, 7). Even among relatives carrying the same mutation (e.g., *MAPT* P301S), clinical presentations can vary, with some individuals exhibiting parkinsonism as the earliest manifestation while others primarily develop frontotemporal dementia (8). In tauopathies, Tau can aggregate in different brain regions and in different cell types; primarily neurons (9), including nigrostriatal neurons (10), but also in glial cells (11, 12), which affects the clinical presentation of these disorders (13). Genome-wide association studies have also found associations between *MAPT* haplotypes with diseases such as PSP (14) and Parkinson’s disease (PD) (15), underscoring the importance of Tau biology for these diseases.

To model the genetic form of FTLD with Tau, transgenic mouse models have been developed (16, 17, 18). A widely used transgenic mouse model is the PS19 line (17), which carries a heterozygous *MAPT* mutation (P301S) (19). *MAPT* P301S mice express human mutant *MAPT* P301S under a mouse prion protein (Prnp) promoter resulting in a five-fold higher expression of human mutant Tau than mouse endogenous protein (17). While phenotypic drift has been reported (20, 21), PS19 mice have originally been described to present neuronal Tau inclusions in the neocortex, amygdala, hippocampus, brain stem, and spinal cord at six months with progressive accumulation over time. Neuronal loss and brain atrophy are present by eight months (17), leading to age-associated cognitive impairment and motor abnormalities (22, 23, 24), followed by limb weakness (17). These motor deficits progress to paralysis at seven to 10 months and significant mortality by 12 months, with median survival of about nine months in male mice, whereas female mice present a survival advantage due to less weight loss and fewer comorbidities (17, 24).

Changes in lipid metabolism have been identified as pathogenic contributor and potential druggable target in multiple neurodegenerative diseases including Alzheimer’s disease (AD) (25, 26), PD (27, 28, 29), and Huntington’s disease (30). For example, in PD, α-synuclein has been shown to interact with membranes and regulate lipid metabolism (31, 32, 33). Recent data also indicates a connection between (mutant) Tau and brain lipid metabolism. In human induced pluripotent stem cell-derived organoids, *MAPT* mutations (namely R406W and V337M) increase cholesterol and fatty acid (FA) synthesis genes in astrocytes (34). In vivo, *MAPT* mutations have been reported to exhibit downregulation of cholesterol synthesis genes in hippocampal astrocytes (35) (in *MAPT* P310S mice) and in hippocampal neurons (36) (in THY-Tau22 mice carrying both *MAPT* G272V and *MAPT* P301S mutations). *MAPT* P301S mutant mice also show decreased expression of cholesterol synthesis proteins in the neurons of spinal cord (37). While these findings in gene and protein expression are indicative of changes in lipids, lipid metabolites themselves were not analyzed in these studies. In another study in THY-Tau22 mice, 24S-hydroxycholesterol levels (a metabolite of cholesterol synthetized by the enzyme CYP46A1) were assessed and shown to be decreased in plasma and cerebrospinal fluid (CSF) (38). Lipidomics studies in brainstem and CSF of *MAPT* mutant rats (expressing human truncated Tau aa151-391/3R) showed changes in glycerolipids and sphingolipids prior to pathogenic Tau accumulation (39), whereas lipid droplets were shown by staining to accumulate with advancing pathology (39). More recently, Tau has been shown to be required for glial lipid droplet formation and turnover in flies and in primary rat astrocytes and human oligodendrocyte-like cells following Tau overexpression or loss of function (40), further implicating Tau as a regulator of brain lipid metabolism.

This effect of Tau on lipid metabolism is notable, as conversely, lipid metabolic processes have also been shown to regulate Tau biology. For example, ApoE4, a major regulator of brain lipid metabolism, exacerbates Tau-mediated neurodegeneration (41, 42) and increasing lipid efflux in ApoE4/Tau transgenic mice by an LXR-agonist inhibits Tau-mediated neurodegeneration and reduces astrocytic and microglial reactivity (43). Inhibition of CYP46A1 activity in the APP23 AD mouse model limits cholesterol export from the brain and increases levels of hyperphosphorylated Tau (pTau) (44), while overexpression of CYP46A1 (to induce export of brain cholesterol) in THY-Tau22 mice rescues Tau-mediated memory impairment (38). Lastly, pharmacological activation of CYP46A1 by the HIV-drug efavirenz reduces phosphorylated Tau levels in human induced pluripotent stem cell-derived neurons (45) and low-dose efavirenz is currently being investigated as a strategic therapy to reduce Tau pathology in AD (46, 47).

To define the effect of *MAPT* mutations and pTau accumulation associated with FTLD on brain lipid metabolism in vivo, we performed lipidomics and sterol analysis in the brains of 9-month-old Tau transgenic mice (*MAPT* P301S, PS19 line). We find that the *MAPT* P301S mutation decreases the levels of triglycerides (TG) in the hippocampus and to lesser extent in the cortex, specifically TG containing 16:1 (palmitoleic acid) and 18:1 (oleic acid) FA. In the hippocampus, we observed increased levels of diglycerides (DG), sphingolipids hexosylceramides and lactosylceramides (HexCER and LacCER), and unsaturated phosphatidylethanolamines (PE). In addition to these differences to the wild-type (WT) group driven by the mutation in *MAPT*, we also observed that within the *MAPT* P301S group, increasing accumulation of pTau in the hippocampus is associated with enrichment in neutral storage lipids (cholesteryl esters, CE and TG) and a decrease in cholesterol synthesis. Overall, our data indicates that the *MAPT* P301S mutation and accumulation of pTau are associated with distinct brain lipidomes in vivo, further implicating altered lipid metabolism as a potential pathogenic and druggable process in primary tauopathies.

## Materials and methods

### Animals

Female WT noncarrier and mice hemizygous for Tg(Prnp-*MAPT*∗P301S)PS19Vle mutation (P301S mice, 008169) were purchased at age of 4–8 weeks from Jackson Laboratories. 16 mice per group were included in the study. The health status was specific pathogen free according to FELASA 2014 recommendations. Upon arrival, the animals were divided into three batches and group housed with two mice per cage and remained group housed in the same social group until the end of the study. The welfare of the animals was maintained according to the European guidelines (EU Directive 2010/63/EU) and Dutch legislation. This included granting of an experiment license by the Centrale Commissie Dierproeven (CCD) under project number AVD112002016590 and approval of the study work protocol by the Instantie voor Dierenwelzijn (IvD).

At 9 months of age, mice were sacrificed and perfused with PBS (VWR) containing heparin (Sigma-Aldrich) and the right hemisphere was immersed in 4% paraformaldehyde (Sigma-Aldrich) for 5 days, after which they were placed in 30% sucrose (Sigma-Aldrich) for future histological analysis. The left hemisphere was freshly dissected, separating hippocampus and cortex, frozen by placement on dry ice, and subsequently stored at −80°C. Plasma samples were collected for sterol measurements.

### Body weight and neurological score

The body weight of each animal was measured longitudinally every 2 weeks, except for weeks 5 and 6, during which the mice were weighted consecutively one week after the other, for the duration of this experiment. General wellbeing of the mice was assessed every two weeks by observation of a number of characteristics. The neurological score is taken from studies in amyotrophic lateral sclerosis mouse models (48). The scoring system was as follows: 0, full extension of hind legs away from lateral midline when mouse was suspended by its tail and mouse could hold this for 2 s, repeated 2–3 times; 1, collapse or partial collapse of leg extension towards lateral midline (weakness) or trembling of hind legs during tail suspension; 2, toes curled under at least twice during walking of 12 inches or any part of the foot is dragging along cage bottom/table (food provided on cage floor); 3, rigid paralysis or minimal joint movement, foot not being used for forward motion; 4, mouse could not right itself within 30 s from either side.

A humane endpoint was defined as a body weight of 80% lower than the highest measured body weight (median of highest three measurements) or a neurological score of 3, indicating motor function problems that could lead to inability to reach food. From the 16 mice per group originally included in the cohort, two mice in the WT group and five mice in the *MAPT* P301S group reached humane endpoint before the end of the study and were excluded from all analysis. (WT n = 14; *MAPT* P301S n = 11).

### Elevated plus maze

Elevated plus maze (EPM) was used as measure of anxiety-related behavior in the study. Mice were introduced onto the center of an EPM facing a closed arm (arms 30 × 6 cm, walls 35 cm high, elevated 50 cm above the ground). The EPM was illuminated with a single white fluorescent light bulb from above (open arms 70 lx, closed arm 30 lx) and exploratory behavior was video tracked for 5 min (Viewer 2, BIOBSERVE GmbH, Bonn, Germany). The border between center and arm entries was defined at 3 cm into each arm. Zone visits were analyzed using the EPM plugin of the tracking software, which was set to count zone visits if both the nose and body reference point had crossed a zone border. The number of open arm visits and total time spent on the open arms were used as readouts.

### Grip strength

Neuromuscular function was assessed from weeks 13–36 of age by sensing the peak amount of force (N) mice applied in grasping a pull bar connected to a force meter (1027DSM Grip Strength Meter, Columbus instruments, Columbus, OH; and Grip Strength Meter 47200, Ugo Basile, Gemonio, Italy). Mice were allowed to grasp the pull bar five times with front paws only, followed by grasping five times with front and hind paws. The median of each five repetitions was taken as grip strength.

### Rotarod

Motor function and motor learning was evaluated from 13 to 36 weeks of age using an accelerating rotarod (Roto-rod series 8, IITC Life Science, Woodland Hills, CA). In the first week of testing, mice received two habituation trials of 120 s (acceleration of from 0 to 20 rpm in 120 s) followed by three test trials (acceleration of from 0 to 40 rpm in 180 s) on the first day and five test trials on the second day. During subsequent testing weeks, mice received 1 day of test trials only with five trials (acceleration of from 0 to 40 rpm in 180 s).

### Morris water maze

Spatial memory was tested in a Morris water maze setup. A circular pool (ø 125 cm) was filled with water (30 cm below the rim) which was painted white with nontoxic paint and kept at a temperature of 25°C. An escape platform (ø 9 cm) was placed at 30 cm from the edge of the pool submerged 1 cm below the water surface. Visual cues were located around the pool at a distance of ∼1.5 m. During testing, lights were dimmed and covered with white sheets and mice were video-tracked using ViewerII (Viewer 2, BIOBSERVE GmbH). Mice were trained for 5 consecutive days, two sessions of two trials per day with a 1–3 min intersession interval. In each trial, mice were first placed on the platform for 30 s and then placed in the water at a random start position and allowed a maximum of 60 s to find the platform. Mice that were unable to find the platform within 60 s were placed back on the platform by hand. Within each 2-trial session, after 30 s on the platform, mice were tested again. On day 5 or 6, a probe trial was performed with the platform removed. Mice were placed in the pool opposite from the platform location and allowed to swim for 60 s. During training trials, the latency, distance, and speed to reach the platform were measured; in the probe trial, the time spent and distance traveled in each quadrant of the pool were measured, as well as the number of platform-zone crossings.

### T-maze

Short-term spatial memory was assessed in a T-maze (white PVC, arms 30 x10 cm) as previously described (49). A sample trial was started by placing a mouse into the start arm (base of the T) to explore the maze. During a sample trial, a removable central partition (17 cm long) protruded from the center of the back wall into the start arm, forcing mice to choose left or right arm while positioned in the start arm. After an entry into a goal arm, a guillotine door at the entrance of the goal arm was lowered, and the mouse was contained in the arm for 30 s. To prepare for the test trial, the central partition was removed and a guillotine door at the end of the start arm was lowered. The test trial was initiated by placing the mouse in the start arm and removing both the guillotine door from the goal arm and the guillotine door at the end of the start arm. The percentage of alternations was used as a readout. A successful alternation was scored if mice choose to enter the previously nonvisited goal arm. A total of six sample and test trials were performed, distributed across 2 days with at least 1 h in between.

### Immunostainings

The right hemisphere of each mouse was embedded in optimal cutting temperature compound cryostat embedding medium (Scigen 4586) and sectioned coronally at 40 μm using a cryostat microtome (Cryostar NX50, Epredia) at −10°C and stored at 4°C in PBS with 0.02% sodium azide (Sigma-Aldrich). For immunohistochemical analysis, three sections from each mouse (300 μm apart), corresponding approximately to bregma coordinates −1.4, −1.7, and −2.0 mm, were used for pTau staining. Brain sections were washed in TBS buffer three times followed by incubation in 0.3% hydrogen peroxide (Sigma-Aldrich) in TBS for 10 min at room temperature. After three washes in TBS, sections were blocked with 3% skim milk powder (Sigma-Aldrich) and 0.25% Triton X-100 (Thermo Fisher Scientific) in 1xTBS for 30 min followed by incubation at 4°C overnight with biotinylated AT8 antibody (1:500, MN1020B, Thermo Fisher Scientific). The following day, the slices were developed using a VECTASTAIN Elite ABC HRP Kit (Vector laboratories) following the manufacturer’s instructions and incubated in a peroxidase substrate solution (DAB, Vector laboratories) for 5 min. Stained sections were imaged with an Olympus VS200 slide scanner, and pathology was quantified using QuPath. In the WT group, samples corresponding to 14 mice were analyzed. In the *MAPT* P301S group, 10 hippocampal samples were analyzed and 11 cortical samples were analyzed (one hippocampal tissue sample was damaged during staining).

For immunofluorescence, two sections (bregma −1.7 mm and −2.0 mm) from each mouse were used. The sections were washed in TBS three times, and blocked with 3% BSA (Sigma-Aldrich) in 0.25% Triton X-100 (Thermo Fisher Scientific) in 1xTBS for 30 min at room temperature, followed by overnight incubation at 4°C with primary antibodies (ADFP: 1:500, EPR3713, Abcam; glial fibrillary acid protein (GFAP): 1:1000, 173004, Synaptic Systems; Iba1: 1:100, ab283346, Abcam). The following day, the sections were washed three times in TBS for 10 min and incubated with fluorescence-labeled secondary antibodies Alexa Fluor (1:1000, Thermo Fisher Scientific) in blocking solution for 2 h at room temperature. The slices were then washed and mounted in ProLong Gold Antifade mounting medium (Molecular Probes). Stained sections were imaged with an Olympus VS200 slide scanner at 40X magnification. Images were deconvoluted using Huygens Professional 21.10 software (scientific volume imaging B.V.) and batch-analyzed using Image J. For representative images of sections in Supplemental Fig. S2, the brightness and contrast values of GFAP and Iba1 staining were adjusted individually to distinguish the signal of interest for each example because of the high variability of background signal between imaged brain slices. Analyses of the images were performed blinded. GFAP- and Iba1-stained images were converted to grayscale 8 bit TIFF file format and preprocessed using non-local means denoising (σ = 15, smoothing factor = 1) and a median filter to reduce speckle noise (“despeckle”) on Iba-1 channel. Regions of interest were determined and particles smaller than 5 pixels were excluded from the mask. The average intensity/pixel values of each area were calculated, and the average intensity/pixel values representing the background intensity were subtracted from those of the immunolabeled areas. The area and intensity of signal within the Iba1 and GFAP masks was measured. In the WT group, samples corresponding to 14 mice were analyzed. In the *MAPT* P301S group, 11 hippocampal samples were analyzed.

### Tissue homogenization

Mouse cortex and hippocampus samples from the left hemisphere were weighted, and stainless steel beads and LC-MS grade water were added to each sample to reach a final concentration of 500 mg/ml. Protease (1:100, Sigma-Aldrich) and phosphatase (1:100, Thermo Fisher Scientific) inhibitors were added to the water to preserve protein content for further analysis. Brain samples were then homogenized using a Next Advance bullet blender. Aliquots were prepared for lipidomics analysis and sterol measurements as described below. The remaining homogenized tissue was stored at −80°C until further use in Western blot analysis.

### Western blot

Homogenized tissue samples (2.5 mg from hippocampus and 15 mg from cortex samples) were resuspended in RIPA buffer (50 mM Tris-Hcl pH 7.4, Roche; 150 mM NaCl, Sigma-Aldrich; 1% NP-40, Thermo Fisher Scientific; 0.5% sodium-deoxycholate, Sigma-Aldrich; 0.1% SDS, VWR; 1 mM EDTA, AppliChem) with the addition of protease (1:100, Sigma-Aldrich) and phosphatase (1:100, Thermo Fisher Scientific) inhibitors and stored at −20°C until further use. Pierce™ BCA Protein Assay Kit (Thermo Fisher Scientific) was used to measure the protein concentration of each sample. Samples were mixed with Laemmli sample buffer 5X (10% SDS, VWR; 50% glycerol, VWR; 312.5 mM Tris pH 6.8, Roche; 0.05% bromophenol blue, AppliChem) with dithiothreitol (250 mM, Thermo Fisher Scientific) to reach a concentration of 2 mg/ml. Samples were denatured at 95°C for 5 min and shortly vortexed. Ten micrograms or twenty micrograms of protein per hippocampus and cortex sample, respectively, were loaded in a 4–15 Criterion™ TGX Stain-Free™ Precast gel (Bio-Rad) as well as 5 μl of Page Ruler Prestained Protein Ladder (Thermo Fisher Scientific). After running the gel (30 min at 90V, followed by 45 min at 150V), proteins were transferred using the Trans-Blot Turbo RTA Midi 0.45 μm LF PVDF Transfer Kit (Bio-Rad) in a BioRad Transblot transfer machine for 7 min at 25V, 2.5 A. The membrane was then incubated in blocking solution with 0.05% Tween-20 (VWR) and 5% BSA (Sigma-Aldrich) in 1xTBS for 30 min at room temperature before being incubated with primary antibodies (AT8: 1:1000, MN1020, Thermo Fisher Scientific; PHF1: 1:500, gifted by P. Davies; total Tau: 1:1000, T6402, Sigma-Aldrich; actin: 1:5000, MAB1501, Merck) overnight at 4°C with gentle shaking. The membrane was washed three times with 0.05% Tween-20 (VWR) in TBS for 5–10 min each wash and incubated with secondary antibodies IRDye® 800CW (1:10,000, LICOR) and/or antibody-conjugated horseradish peroxidase (Dako) for 1 h at room temperature with gentle shaking. After the incubation, the membrane was washed for 5 min at room temperature three times with 0.05% Tween-20 (VWR) in TBS. Membranes were scanned using the LI-COR® Odyssey® Fc Imaging System (LI-COR, Cambridge, UK). For chemiluminescence imaging, luminol-based enhanced chemiluminescence horseradish peroxidase substrate Thermo Scientific™ SuperSignal™ West Dura Extended Duration Substrate (Thermo Fisher Scientific) was added on the membrane. Image StudioTM Lite 5.2.5 Software (LI-COR) was used to quantify band signal. In the WT group, 11 cortical samples and one hippocampal sample (run on the same lane as the molecular weight marker) were analyzed. In the *MAPT* P301S group, 11 hippocampal samples and 10 cortical samples were analyzed (one cortical sample was lost during preparation). Image brightness and contrast were adjusted for optimal visualization of the bands.

### Sterol measurements

Cholesterol, noncholesterol sterols, and oxysterols were quantified in plasma, cortex, and hippocampus homogenates from n = 14 WT mice and n = 11 *MAPT* P301S mice. To 150 μl plasma, to 2.5–10 mg hippocampus, and 25 mg cortex homogenates, 5α-cholestane, epicoprostanol, and deuterium-labeled 7α-, 24-, and 27-hydroxycholesterol were added as internal standard. After alkaline hydrolysis, cholesterol and noncholesterol sterols—including cholesterol surrogate absorption markers: 5α-cholestanol, campesterol, sitosterol, brassicasterol, stigmasterol, 5α-campestanol, and 5α-sitostanol; cholesterol surrogate synthesis markers: lathosterol, desmosterol, lanosterol, 23,24-dihydrolanosterol; and cholesterol oxysterols and bile acid precursors: 7α-, 24S- and 27-hydroxycholesterol—were extracted by cyclohexane. After silylation of the free hydroxyl groups to trimethylsilyl ether, sterols and oxysterols were measured by gas chromatography-mass spectrometry-selected ion monitoring as previously described (50).

Cholesterol and 5α-cholestanol were detected by flame ionization detection using 5α-cholestane as an internal standard (ISTD). The noncholesterol sterols (epicoprostanol, ISTD) and the oxysterols (^2^H_x_-oxysterols, ISTD) were detected by a highly specific and sensitive quadruple mass spectrometer (HP5975B inert mass selective detector, Agilent Technologies, Waldbronn, Germany) operated in selected ion monitoring mode (MS-SIM). Gas chromatographic separation and detection of cholesterol, 5α-cholestanol, and 5α-cholestane (ISTD) were performed on a DB-XLB column with a 30 m × 0.25 mm i.d. × 0.25 μm film (J&W Scientific Alltech, Folsom, CA) in a Hewlett-Packard (HP) 6890 series GC system (Agilent Technologies, Palo Alto, CA), equipped with a flame ionization detector (FID).

Noncholesterol sterols and authentic and deuterium-labeled oxysterols were separated on another DB-XLB column with a 30 m × 0.25 mm i.d. × 0.25 μm film (J&W Scientific Alltech) in an HP 6890N Network GC system (Agilent Technologies) connected with a direct capillary inlet system to an inert quadruple mass selective detector HP5975B (Agilent Technologies). Both GC systems were equipped with HP 7687 series automatic samplers and HP 7683 series injectors (Agilent Technologies). To determine the concentrations of cholesterol in brain and plasma, its main steroidal precursors, plant sterols, and oxysterols, 50 μg of 5α-cholestane (Serva, Heidelberg, Germany) (50 μl from a stock 1 mg/ml solution of 5α-cholestane in cyclohexane obtained from Merck KGaA, Darmstadt, Germany), 1 μg of epicoprostanol (Sigma, Deisenhofen, Germany) (10 μl from a stock 100 μg/ml solution of epicoprostanol in cyclohexane); and 50 ng of racemic [23,23,24,25-^2^H_4_]24(R,S)-hydroxycholesterol (Medical Isotopes, Pelham, NH); 100 ng 26,26,26,27,27,27-[^2^H_6_]-7α-hydroxycholesterol; and 100 ng [16,16,17,20,22-^2^H_5_]-(25R)27-hydroxycholesterol (Medical Isotopes) (50 μl from a 2 μg/ml stock solution in toluene obtained from Merck KGaA, Darmstadt, Germany) were added as internal standards to 150 μl plasma or to a chloroform/methanol tissue extract (5 ml chloroform/methanol, 2:1, v/v, per 10 mg brain tissue). To avoid autoxidation, 50 μl of a 2,6-di-tert-butyl-4-methylphenol/methanol solution (Sigma-Aldrich Chemie GmbH, Taufkirchen, Germany) were added. After saponification with 2 ml of 1 M 95% ethanolic sodium hydroxide solution (Merck KGaA, Darmstadt, Germany) at 60°C for 1 h, the free sterols and oxysterols were extracted three times with 3 ml of cyclohexane each. The organic solvent was evaporated by a gentle stream of nitrogen at 60°C on a heating block. The residue was dissolved in 80 μl n-decane (Merck KGaA). An aliquot of 40 μl was incubated (1 h at 70°C on a heating block) by the addition of 20 μl of trimethylsilylating (TMSi) reagent—comprising chlorotrimethylsilane (Merck KGaA), 1,1,1,3,3,3-hexamethyldisilasane (Sigma-Aldrich, St. Louis, MO), and pyridine (Merck KGaA, 9:3:1)—in a GC vial for GC–mass selective detector noncholesterol and oxysterol analysis. Another aliquot (40 μl) was incubated by the addition of 40 μl of the TMSi reagent and dilution with 300 μl of n-decane in a GC vial for GC–FID cholesterol analysis. An aliquot of 2 μl was injected by automated injection in splitless mode using helium (1 ml/min) as the carrier gas for GC–MS-SIM and hydrogen (1 ml/min) for GC–FID analysis at an injection temperature of 280°C. The temperature program for GC was such that it was 1) kept at 150°C for 3 min, 2) heated at 20°C/min, and 3) kept at 290°C for 34 min. For mass selective detection, electron-impact ionization was applied with 70 eV. MS-SIM was performed by cycling the quadruple mass filter between different *m/z* values at a rate of 3.7 cycles/s. The TMSi derivatives of the noncholesterol sterols and the di-TMSi derivatives of the oxysterols were monitored using the following: cholesterol at *m/z* 458; 5α-cholestanol at *m/z* 460; lathosterol at *m/z* 458 (M^+^); desmosterol at *m/z* 441 (M^+^ −15, M^+^ -CH_3_); lanosterol at *m/z* 393 (M^+^ -90–15, M^+^ -OTMSi-CH_3_); 22,23-dihydrolanosterol at *m/z* 395 (M^+^ -90–15, M^+^ -OTMSi-CH_3_); campesterol at *m/z* 472 (M^+^); sitosterol at *m/z* 486 (M^+^); stigmasterol at 484 (M+); brassicasterol at 470 (M+); campestanol at *m/z* 474 (M^+^); sitostanol at *m/z* 488 (M^+^); 26,26,26,27,27,27-[^2^H_6_]-7α-hydroxycholesterol at *m/z* 462 (M^+^ −90, M^+^-OTMSi); 7α-hydroxycholesterol at *m/z* 456 (M^+^ −90, M^+^-OTMSi); [23,23,24,25-^2^H_4_]24(R,S)-hydroxycholesterol at *m/z* 416 (M^+^ -90–44, M^+^ -OTMSi-CD(CH_3_)_2_); 24(S)-hydroxycholesterol at *m/z* 413 (M^+^ -90-43, M^+^ -OTMSi-CH(CH_3_)_2_); [16,16,17,20,22- ^2^H_5_]- (25R)27-hydroxycholesterol at *m/z* 461 (M^+^ −90); and (25R)27-hydroxycholesterol at *m/z* 456 (M^+^ −90). Peak integration was performed manually. Cholesterol and 5α-cholestanol in plasma were directly quantified by multiplying the ratios of the area under the curve of cholesterol or cholestanol to 5α-cholestane by 50 μg (ISTD amount). Noncholesterol sterols and oxysterols in plasma and homogenates and cholesterol and 5α-cholestanol together with noncholesterol in homogenates were quantified using the ratios of the areas under the curve of the respective noncholesterol sterols/oxysterols after MS-SIM analyses against internal standards were performed using standard curves for the listed sterols/oxysterols. Identification of all sterols was verified by comparison with the full-scan mass spectra of authentic compounds. Additional qualifiers (characteristic fragment ions) were used for structural identification (*m/z* values not shown). Four WT mice were excluded from the total cholesterol assessment due to insufficient sample availability. Sterols, oxysterols, and phytosterols were normalized to total cholesterol to normalize for different input.

### Lipidomics analysis

Aliquots from hippocampus and cortex homogenates of 14 WT mice and 11 *MAPT* P301S mice containing the equivalent of 5 mg of tissue per sample were used for lipidomics analysis. Lipidomics analysis followed standardized, quantitative protocols (51). Briefly, 25 μl Lipidyzer internal standard mix containing 54 deuterated standards was added to 5 mg of tissue and extraction followed a methyl tert-butyl ether-based protocol. After drying under a gentle stream of nitrogen, samples were dissolved in running buffer (methanol:dichloromethane 1:1, containing 10 mM ammonium acetate) and injected into the Lipidyzer platform, consisting of a SCIEX QTRAP 5500 mass spectrometer equipped with SelexION DMS interface and a Nexera X2 UHPLC- system. Shotgun Lipidomics Assistant (SLA) software was used to process data files and report the lipid class and species concentration and composition values (52). SLA analyzes DMS lipidomics data to give quantitative lipid species results. It does so by importing mzML files that are being extracted from the WIFF files originating from the Sciex Analyst software using Proteowizard. Together with the mzML files, the species name dictionary, standard dictionary, and isotope correction list are imported as xls files into the software. The functions of these files are 1) assigning lipid species names to the data in the mzML file, 2) assigning internal standard and its concentration to quantify the lipid species, and 3) correct for isobaric overlap within the data. Together, this generates two results files, one for method 1 and for method 2. As a final step, these files are combined with a sample file that contains specific normalization factor. The result is an excel file with quantitative lipid data, ready for further data analysis. Lipidyzer data analysis was further accomplished using SODAlight as a built-in data browser for the Neurolipid atlas repository (53). Lipid species concentration datasets were imported and filtered, with individual species required to have a minimal intensity of two times the blank in at least 80% of all samples measured. If lipid species were absent or below two times the blank in >20% of all samples, they were removed. No missing value imputation was done. The SLA control software, including all up to date dictionaries and isotope correction algorithms can be found here [https://github.com/syjgino/SLA]. SODA-light is a development branch of iSODA [https://github.com/ndcn/soda-ndcn] and part of the Neurolipid Atlas. The code for SODA light is available at [https://github.com/CPM-Metabolomics-Lipidomics/soda-light]. SODA light version 0.2 was used for the generation of lipidomics figures in this manuscript. Raw data in nmol/g (used for genotype comparisons) and values normalized over total lipid concentration (used for brain region comparisons, due to hippocampal and cortical samples being processed on separate lipidomics batches) will be added to the Neurolipid Atlas repository [https://neurolipidatlas.com/].

### PE and TG saturation analysis

To investigate differences in the saturation of lipid classes between groups, the sum of the concentration of the lipid species with identical number of double bonds within the TG or within PE was calculated. Afterward, the fold change from each sample was calculated over the mean of the control samples.

### Statistics

Statistical analysis was performed in Graphpad Prism version 10.3.1 (GraphPad Software, Boston, MA). Gaussian distribution was evaluated using the D’Agostino & Pearson normality test. The statistical tests used and the sample size (n) are annotated in the figure legends. Correlation between pTau epitopes and lipid classes, lipid species, and sterols was computed as Pearson correlation coefficient when data followed a normal distribution or as Spearman correlation coefficient when data was nonparametric (indicated in the figure legends). Correlation coefficients from lipid species that were detected in less than half of the samples were excluded from visualization.

## Results

### Behavioral and pathological characterization of *MAPT* P301S mice shows variability across aged-matched mice

**Fig. 1:**
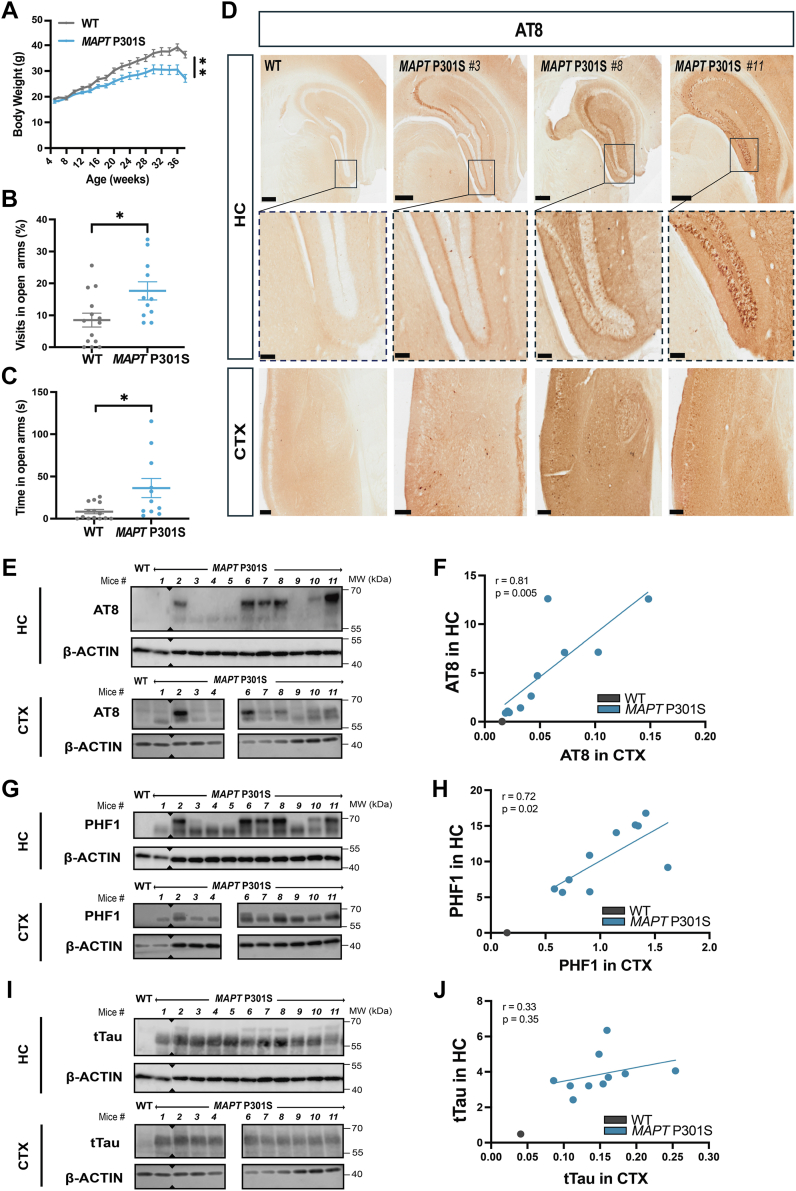
Behavioral and pathological characterization of MAPT P301S female mice. A: Body weight trajectories of WT (n = 14) and MAPT P301S (n = 11) mice from weeks 4–38 of age. Two-way ANOVA test was applied. B and C: Elevated plus maze test at 13 weeks of age analyzing percentage of visits to open arms (B) and time spent in open arms (C) of WT (n = 14) and MAPT P301S (n = 11) mice. Student’s *t* test was applied. D: Representative immunohistochemistry images of AT8-stained hippocampus (HC) and cortex (CTX) of WT (n = 14) and MAPT P301S (HC n = 10; CTX n = 11) mice at 9 months of age. Mice genotype and mice identifier number are indicated. Also labeled is dentate gyrus (DG) and CA1 region of the hippocampus. Scale bars: 400 μm HC, 100 μm DG, 200 μm CTX. E–J: Western blot images of AT8 (E, quantified in F), PHF1 (G, quantified in H), total Tau (tTau) (I, quantified in J), and beta actin (β-actin) levels in lysates from HC (n = 11) and CTX (n = 10) from a representative WT mouse example and MAPT P301S mice at 9 months of age. AT8, PHF1 and tTau quantifications are relative to β-actin levels. Sections where the blots were cut are indicated with triangles. AT8, tTau, and β-actin were analyzed on the same blot and therefore the same β-actin blot image is shown both for E and I. Correlation values between HC and CTX levels of pTau and tTau within the MAPT P301S mice are indicated as Pearson’s correlation coefficient (r) and *P* values. Error bars represent mean ± SEM. Each data point represents one individual mouse; MAPT P301S mice are depicted in blue, WT mice are depicted in *gray*. ∗*P* < 0.05, ∗∗*P* < 0.01.

To study how *MAPT* mutations affect brain lipid metabolism, we aged a cohort of female WT and *MAPT* P301S mice to 9 months, after which a panel of analyses and motor and behavioral tests were performed to evaluate the disease status of our cohort (Fig. 1A–C, Supplemental Fig. S1). As reported before, female *MAPT* P301S mice showed lower body weight and reduced anxiety than WT mice (Fig. 1A–C) but did not show deficits in cognition (Supplemental Fig. S1A–C). Unexpectedly, we did not find motor impairments in our cohort of *MAPT* P301S mice even at 9 months of age (Supplemental Fig. S1D, E). We therefore assessed the extent of Tau pathology in the brains of the mice in this cohort. We determined the levels of pTau in *MAPT* mice by immunohistochemistry (Fig. 1D) with AT8 staining, which measures phosphorylation at serine 202 and threonine 205 (S202/T205). In addition, by Western blot (WB) (Fig. 1E–H), we analyzed levels of PHF1, which measures phosphorylation at serine residues 396 and 404 (S396/S404), and AT8 reactive Tau, in cortical and hippocampal homogenates. By immunohistochemistry, AT8 staining was absent in WT mice (Fig. 1D); whereas in *MAPT* P301S mice, AT8 staining was highly variable, ranging from mice that showed no staining to strong signal in extended areas of the cortex and the hippocampus (Fig. 1D). This variability was also reflected by WB analysis, where we observed mice with low and other mice with very high levels of AT8 and PHF1 staining within the transgenic mice group in both the cortex and the hippocampus (Fig. 1E–H). A higher molecular weight species of Tau (as assessed by total Tau staining) was also observed in mice with high pTau levels (Fig. 1I, Supplemental Fig. S1F). As expected, the transgene increased Tau levels in *MAPT* P301S, whereas total Tau (with the exception of the higher molecular weight band) was relatively similar among mice within the *MAPT* P301S group (Fig. 1I, J). Another key hallmark of Tau transgenic mice is the occurrence of astrogliosis and microgliosis. By staining for GFAP and Iba1, respectively, we also observed increased astrogliosis and microgliosis in mice with the highest levels of pTau (Fig. S2). Overall, our behavioral and biochemical characterization show heterogeneity in disease stages even between mice of the same age, where pTau accumulation is absent in some mice whereas others have more advanced pTau accumulation and gliosis.

### Mouse brain lipidomics reveals distinct hippocampal and cortical lipid composition

**Fig. 2:**
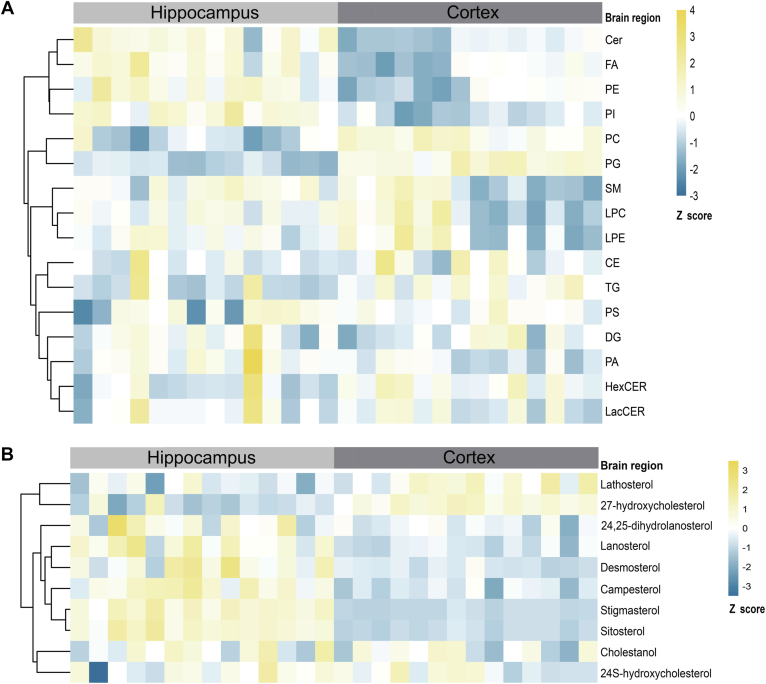
WT mouse brain lipidomics across hippocampal and cortical brain regions. A and B: Heatmap shows Z-scored relative lipid class (A) and sterol (B) abundance in WT mice (n = 14) in hippocampal (HC) and cortical (CTX) brain regions at 9 months of age. Lipid classes analyzed include the following: cholesteryl ester (CE), ceramide (Cer), diglyceride (DG), fatty acid (FA), hexosylceramide (HexCER), lysophosphatidylcholine (LPC), lysophosphatidylethanolamine (LPE), lactosylceramide (LacCER), phosphatidic acid (PA), phosphatidylcholine (PC), phosphatidylethanolamine (PE), phosphatidylglycerol (PG), phosphatidylinositol (PI), phosphatidylserine (PS), sphingomyelin (SM), triglyceride (TG).

To understand how *MAPT* mutations might affect brain lipid metabolism—possibly in a brain region-specific manner—we performed widely targeted lipidomics on hippocampus and cortex, studying 16 different lipid classes and over 1,400 lipid species. We also separately measured a panel of 10 sterols. Regional variation in the lipid profile across different brain areas has been reported in the mouse brain (54) and indeed our data also revealed differences in lipid profile among these two brain regions in WT mice (Fig. 2A). For example, we observed enrichment of ceramides (Cer) and FA in the hippocampus. Some phospholipids such as PE and phosphatidylinositol showed enrichment in the hippocampus, whereas others such as phosphatidylcholine and phosphatidylglycerol (PG) were enriched in the cortex (Fig. 2A). When exploring regional differences in sterol abundance, we observed that the mouse cortex presented higher levels of lathosterol and 27-hydroxycholesterol than the mouse hippocampus (Fig. 2B). Conversely, the hippocampus was enriched in lanosterol and desmosterol (cholesterol precursors) as well as in campesterol, stigmasterol, and sitosterol (phytosterols) (Fig. 2B). These data indicate that different brain regions are different in terms of lipid profile, which might affect the interplay between Tau and lipid metabolism.

### Effects of *MAPT* P301S mutation on the brain lipidome

**Fig. 3:**
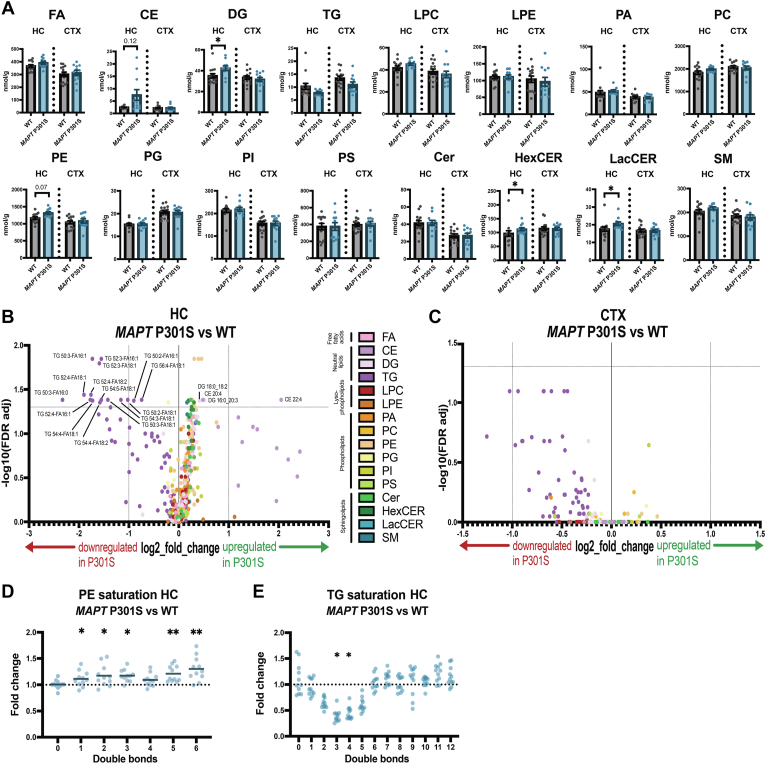
Effects of MAPT P301S mutation on the brain lipidome of 9-month-old mice. A: Changes in all 16 analyzed lipid classes in WT versus MAPT P301S mice in hippocampus (HC) and cortex (CTX). Each data point represents one individual mouse; WT mice are depicted in *gray*, MAPT P301S mice are depicted in *blue*. Error bars represent mean ± SEM. Lipid classes analyzed include the following: cholesteryl ester (CE), ceramide (Cer), diglyceride (DG), fatty acid (FA), hexosylceramide (HexCER), lysophosphatidylcholine (LPC), lysophosphatidylethanolamine (LPE), lactosylceramide (LacCER), phosphatidic acid (PA), phosphatidylcholine (PC), phosphatidylethanolamine (PE), phosphatidylglycerol (PG), phosphatidylinositol (PI), phosphatidylserine (PS), sphingomyelin (SM), triglyceride (TG). B and C: Volcano plot of altered lipid species in MAPT P301S versus WT mice in hippocampus (B) and cortex (C). Significant neutral lipid species (CE, DG, TG) are labeled. D and E: Relative fold change in PE (D) and TG (E) with indicated number of double bonds (unsaturation) in the hippocampus of MAPT P301S versus WT mice. Each data point represents one individual mouse. n = 14 WT, n = 11 MAPT P301S. Mann-Whitney U test with FDR Benjamini-Hochberg correction for multiple comparisons was applied in all subfigures; ∗*P* < 0.05, ∗∗*P* < 0.01.

To understand the effect of *MAPT* P301S mutation on brain lipid composition in the different brain regions, we first compared the abundance of 16 lipid classes between WT and mutant mice in hippocampus and cortex (Fig. 3A). We observed significant changes in the hippocampus, but not in the cortex. In the hippocampus of *MAPT* P301S mice, HexCER and LacCER, as well as DG, were increased. We also observed a trend in the hippocampus towards increased levels of PE in *MAPT* P301S mice (Fig. 3A). While CE (storage form of cholesterol) were not significantly changed, we noticed a high spread in this lipid class across *MAPT* P301S mice in the hippocampus (but not in the cortex), which we further investigated in relation to pTau levels later in the manuscript.

In addition to studying differences at the lipid class level, we also assessed differences in individual lipid species in hippocampus and cortex using a volcano plot (Fig. 3B, C). We found that several PE (PE 16:0_18:2, PE 18:0_18:2, PE-P 18:1/20:4, PE 18:1_20:4, PE-P 18:1/22:1, PE-P 18:0/20:4, PE-P 16:0/20:2, PE 18:1_18:2, and PE-P 18:1/20:5) were significantly upregulated in the hippocampus of mutant mice (Fig. 3B, Supplemental Fig. S3A). Several DG species (DG 16:0_20:3 and DG 18:0_18:2) and HexCER species (HexCER d18:1/22:0, HexCER d18:1/24:0, HexCER d18:1/16:0, and HexCER d18:1/18:0) were also upregulated in the hippocampus of mutant mice as were some other lipids including Cer d18:1/20:1, PG 18:1_20:4, SM d18:1/24:1, and PS 18:0_18:2 (Fig. 3B). Strikingly, multiple TG were significantly downregulated in the hippocampus and showed a similar trend in the cortex of mutant mice (Fig. 3B, C).

To further explore saturation effects in PE and TG, we categorized PE and TG species based on their degree of unsaturation (number of double bonds) and found an overall increase of unsaturated PE in the *MAPT* P301S mutant mice in the hippocampus, but not in the cortex (Fig. 3D, Supplemental Fig. S3B). TG species containing a total of two to five double bonds were significantly decreased in the hippocampus (Fig. 3E) reflecting the decrease in TG containing monounsaturated FA (specifically 16:1 and 18:1) observed at the species level (Fig. 3C). A similar trend was observed in the cortex (Supplemental Fig. S3C), indicating a specific effect of *MAPT* P301S on these TG species. Lastly, at the species level in the hippocampus, we observed a striking shift of most CE species with a large fold change but relatively low significance (Fig. 3B) and two significantly increased CE species (CE 22:4 and CE 20:4). This indicates that some mice have very strongly upregulated CE levels, whereas the effect is absent in other mice.

Overall, these data indicate that the *MAPT* P301S mutation strongly alters TG metabolism in the hippocampus and to a lesser extent in the cortex. The *MAPT* mutation also significantly increases unsaturated PE in the hippocampus specifically, as well as DG and the sphingolipids HexCER and LacCER. Furthermore, a subset of *MAPT* P301S mice has increased levels of CE, which is explored in the context of pTau accumulation later in this manuscript.

### Effects of *MAPT* P301S mutation on brain sterol levels

**Fig. 4:**
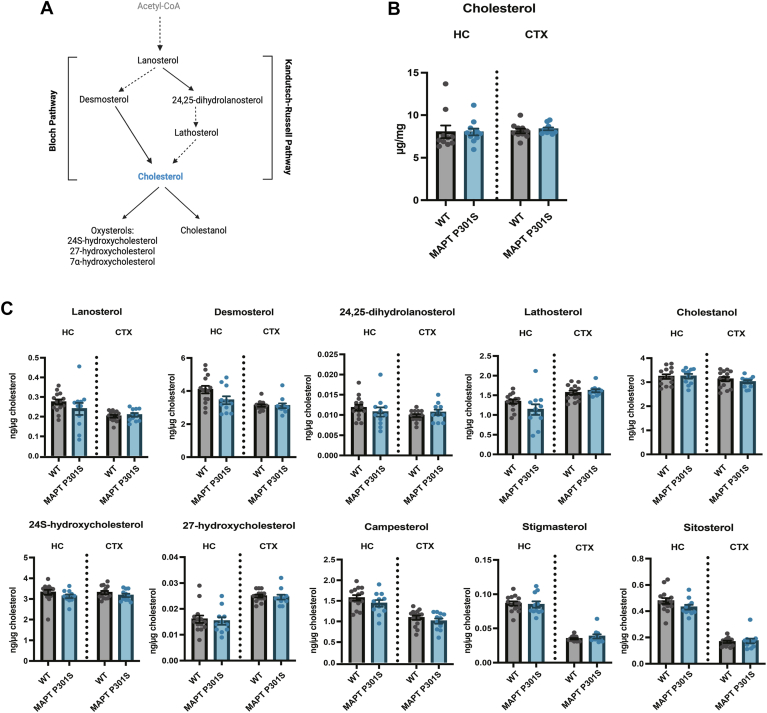
Analysis of sterols, oxysterols, and phytosterols in the brain of 9-month-old MAPT P301S mice. A: Simplified schematic of cholesterol synthesis indicating the measured species. The pathway is separated into two branches (Bloch and Kandutsch-Russell) that converge in cholesterol. Dashed lines indicate omitted intermediate species in the pathway; solid lines indicate a direct conversion. B: Analysis of total cholesterol in MAPT P301S (*blue*; n = 11) and WT (*gray*; n = 10) mice in brain hippocampus (HC) and cortex (CTX). C: Analysis of sterols, oxysterols, and phytosterols normalized to total cholesterol in MAPT P301S (*blue*; n = 11) and WT (*gray*; n = 14) mice in brain hippocampus and cortex. Error bars represent mean ± SEM. Each data point represents one individual mouse. Mann-Whitney test with FDR Benjamini-Hochberg correction for multiple comparisons was applied with a probability level set at 95%.

A number of previous studies have found transcriptomic and proteomic changes in cholesterol synthesis pathways in Tau transgenic mice (35, 37). While CE is included in our standard lipidomics panel, cholesterol and other related sterols are not. Therefore, we extended our analysis by also quantifying several sterols, oxysterols, and phytosterols (the latter derived from diet) in the hippocampus and the cortex of WT and *MAPT* P301S mutant mice (Fig. 4A). Total cholesterol was not different between WT and mutant mice and was used to normalize the levels of other sterols (Fig. 4B). We observed no significant differences in any of the measured sterols between WT and mutant mice in either the cortex or the hippocampus. (Fig. 4C). We also measured cholesterol and other sterols in plasma (Supplemental Fig. S4A, B) and found that *MAPT* P301S mice had significantly lower levels of total cholesterol in plasma (Supplemental Fig. S4A) and significantly reduced levels of 24,25-dihydrolanosterol and cholestanol (Supplemental Fig. S4B). Overall, our data indicates that the *MAPT* P301S mutation does not strongly affect brain sterol levels but, as for CE, we found a large spread in sterols, oxysterols, and phytosterols in the hippocampus of *MAPT* P301S mice that might be associated with different levels of pTau (explored below).

### Accumulation of pTau in the hippocampus is associated with altered cholesterol metabolism and increased neutral storage lipids

**Fig. 5:**
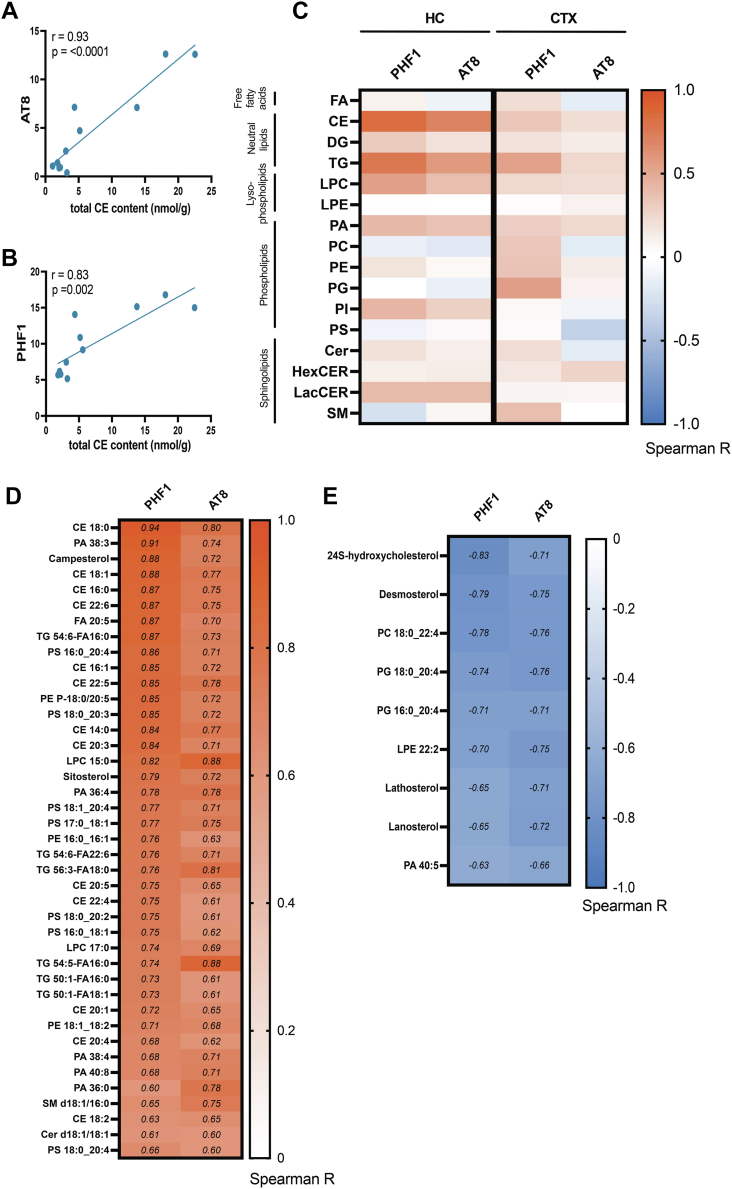
Correlation of pTau accumulation in the MAPT P301S mouse hippocampus with brain lipid and sterol signatures. A and B: Linear correlations between cholesteryl ester (CE) at the class level and relative abundance of pTau epitopes AT8 (A) and PHF1 (B) as determined by Western blot in the hippocampus of MAPT P301S mice. Each data point represents one individual mouse. Pearson’s correlation coefficient (r) is shown for each comparison and *P* value is presented with a probability level set at 95%. C: Heatmap showing Spearman correlation coefficient (r) of all lipid classes with pTau AT8 and PHF1 in hippocampus (HC) and cortex (CTX). Lipid classes analyzed include the following: cholesteryl ester (CE), ceramide (Cer), diglyceride (DG), fatty acid (FA), hexosylceramide (HexCER), lysophosphatidylcholine (LPC), lysophosphatidylethanolamine (LPE), lactosylceramide (LacCER), phosphatidic acid (PA), phosphatidylcholine (PC), phosphatidylethanolamine (PE), phosphatidylglycerol (PG), phosphatidylinositol (PI), phosphatidylserine (PS), sphingomyelin (SM), triglyceride (TG). D and E: Heatmap showing Spearman correlation coefficient (r) > 0.60 of the highest positively correlating (D) and (r) < -0.60 of the highest negatively correlating (E) lipid species (from lipidomics and sterol panel) with relative abundance of pTau AT8 and PHF1 epitopes in the hippocampus of MAPT P301S mice. MAPT P301S n = 11 HC, n = 10 CTX.

As mentioned above, we observed a large spread in CE (Fig. 3A, B) and cholesterol precursors and its metabolites (Fig. 4C) in the *MAPT* P301S mice, which also display a large variation in pTau accumulation (Fig. 1). Therefore, within the *MAPT* P301S group, we tested whether the levels of specific lipid classes (Fig. 5A–C) or species (Fig. 5D–E) correlated with AT8 and PHF1 pTau levels. First, at the class level, we observed a strong positive correlation between CE and pTau in the hippocampus, but not in the cortex (Fig. 5A–C). TG also exhibited a positive correlation with pTau accumulation in both brain regions (Fig. 5C), with the strongest association observed in the hippocampus, indicating that storage lipids increase when pTau accumulates. At the species level (Fig. 5D, E, Supplemental Fig. S5A, B), we found that all of the detected CE species in the hippocampus strongly correlated with increased pTau levels (Spearman r > 0.60) (Fig. 5D). Additionally, many TG species correlated positively with pTau accumulation in this brain region (Fig. 5D), as well as in the cortex with PHF1 (Supplemental Fig. S5A). Multiple other individual lipid species were positively correlated with pTau in the hippocampus, but clear group or saturation effects were not directly obvious. Surprisingly, campesterol and sitosterol (phytosterols derived from diet) showed a strong positive correlation with pTau (Fig. 5D). Campesterol levels also correlated positively with pTau in the cortex (Supplemental Fig. S5A). Lipids that negatively correlated with pTau levels included phospholipids from diverse classes (phosphatidylcholine 18:0_22:4, PG 18:0_20:4, PG 16:0_20:4, and PA 40:5) and LPE 22:2 (Fig. 5E). Cholesterol precursors (lanosterol, desmosterol, and lathosterol) were also negatively correlated with increased pTau in the hippocampus (Fig. 5E), but not in the cortex (Supplemental Fig. S5B), and 24S-hydroxycholesterol correlated negatively with pTau in the hippocampus and to a lesser extent in the cortex (Fig. 5E, Supplemental Fig. S5B–D). To visualize these changes better, we divided the *MAPT* P301S mice in two groups, one with low pTau levels (mice #1, 3–5, 9) and one with high pTau levels (mice #2, 6–8, 10–11) assessed by the relative expression of AT8 and PHF1 epitopes by WB (Fig. 1E, G). Levels of cholesterol precursors were identical between WT and *MAPT* P301S mice with low pTau, whereas levels of such precursors (lanosterol, desmosterol, and lathosterol) as well as 24-hydroxcycholesterol all trended down in the high pTau group (Supplemental Fig. S6A). Overall, these data indicate that pTau accumulation correlates with increased levels of neutral storage lipids (CE and TG) and reduced cholesterol precursors and metabolites (24S-hydroxycholesterol) in the hippocampus.

## Discussion

Lipids are implicated in the pathogenesis of a number of neurodegenerative diseases (25, 26, 27, 28, 29, 30, 55), but few reports have explored how *MAPT* mutations, known to cause parkinsonism and FTLD, affect brain lipid metabolism. Therefore, here we investigated *MAPT* mutation- and pTau-dependent lipid changes by widely targeted lipidomics and sterol analysis in the hippocampus and cortex of *MAPT* P301S mice. We found that, when comparing WT and mutant mice, the *MAPT* P301S mutation was associated with increased levels of DG, sphingolipids HexCER and LacCER, and unsaturated PE in the hippocampus (but not in the cortex). In both hippocampus and cortex, the *MAPT* P301S mutation affects TG metabolism, specifically lowering TG species containing monounsaturated FA, while cholesterol and its metabolites were unchanged when comparing WT and mutant mice at the group level. Within the *MAPT* P301S group, we observed a strong correlation between pTau accumulation and changes in brain lipid metabolism. We found that accumulation of pTau in the hippocampus correlated with increases in neutral storage lipids, such as CE and TG, and a decrease in cholesterol precursors and 24S-hydroxycholesterol. Overall, our data indicates that *MAPT* mutations and pTau accumulation are associated with distinct hippocampal lipidome and cholesterol metabolism in vivo.

Surprisingly, we did not find motor impairments in our *MAPT* P301S mice at 9 months of age. Severe motor deficits have previously been reported at this age for female *MAPT* P301S mice (23), which may suggest a phenotypic drift with delayed onset of pathology (20, 21) or potentially reduced motor manifestations in female mice (24). pTau accumulation has been observed in female *MAPT* P301S mice as early as 3 and 6 months of age for S404 and S396 epitopes (PHF1), respectively. For AT8, pTau has been detected at 3 months of age specifically in the hippocampus, compared to 9 months of age for the rest of the brain (24). Our cohort of 9-month-old *MAPT* P301S showed a large variation in the amount of pTau accumulation, which strongly correlated to lipid metabolic changes, particularly in neutral lipids. We also found that *MAPT* mutations and pTau accumulation exert a more prominent effect in the hippocampus than in the cortex. While we did not directly compare Tau levels between hippocampus and cortex, a higher dependency on Tau function or differential onset of accumulation of pTau in these areas could explain why the hippocampus is more affected. Alternatively, the differences in lipid composition of these areas might determine their response to mutant Tau or pTau accumulation. For example, larger changes in HexCER and LacCER in the hippocampus compared to cortex of *MAPT* P301S mice could be explained by a higher pool of Cer present in the hippocampus as shown in our data. A similar observation can be made regarding hippocampal sensitivity to changes in DG, which could be explained by the hippocampal enrichment of phosphatidylinositol shown in our data, as these phospholipids can be directly converted to DG through enzymatic cleavage. These and other regional differences could also be influenced by cell-type distribution (56) or local cholesterol synthesis enzyme expression (25).

The *MAPT* P301S mutation, independent of the levels of pTau accumulation, was associated with increased HexCER and LacCER in the hippocampus. These glycosphingolipids are synthesized in the Golgi from ceramide by the addition of a glucose or galactose group (HexCER) or both (LacCER). Glycosphingolipids are organized into clusters known as “lipid rafts” in the plasma membrane, where they serve as structural components and support cell signaling functions both outside and within the cell (57). Consistent with our findings, increased levels of HexCER have been found in the CSF of a rat model of tauopathy (39) and increased HexCER and LacCER levels have been reported in the brain of AD patients (53, 58, 59, 60). Changes in these lipids have been associated with neuroinflammation (61, 62, 63) and have been reported to be increased by microglial activation through upregulation of sphingosine kinase, which is involved in the synthesis of these ceramides (64). Another group of lipids altered by the *MAPT* P301S mutation included PE, a major membrane component and an essential mitochondrial membrane phospholipid (65). We found PE to be increased in the hippocampus, in line with what has been reported in the brain tissue of Tau transgenic rats (39). At the same time, we found that the *MAPT* P301S mutation increased DG levels but reduced TG levels. This might indicate that the mutation in *MAPT* causes the diversion of DG toward PE synthesis (through the Kennedy pathway) rather than TG formation, reducing TG output and therefore lipid storage. A metabolic shift away from lipid storage (TG) toward membrane maintenance or repair (PE) may reflect mitochondrial stress or attempts to compensate for it (66, 67). Additionally, PE has been shown to stimulate Tau phosphorylation (68), while in mild but not in severe cases of AD, PE are increased (60).

In addition to the effects of mutant Tau, we also observed a correlation between buildup of pTau and the accumulation of neutral lipids (CE and TG). While we cannot conclude that the accumulation of pTau directly drives this effect, it is plausible that the known role of the P301S mutation on pTau accumulation, or secondary events triggered by pTau accumulation, contribute to this phenotype. Interestingly, CE accumulation has been shown to enhance Tau pathology (45). Additionally, TGs are increased in serum and brain samples of FTLD patients with Tau pathology compared to controls (69) and CE are elevated in the frontal cortex of AD patients with Tau pathology (53). TG and CE are stored in lipid droplets, which numbers are increased in the other rat models of tauopathy (39) and in microglia of individuals with primary tauopathies (PSP, corticobasal degeneration) (39). It is not known whether lipid droplets levels in this context are protective (70) or whether their accumulation is detrimental (71, 72, 73). Nonetheless, previous reports show that lipid droplet-accumulating microglia present decreased phagocytosis and increased levels of proinflammatory cytokines (72, 73), which contributes to neuroinflammation.

Lastly, we observed that pTau accumulation correlated negatively with the levels of cholesterol precursors (lanosterol, desmosterol, and lathosterol) and 24S-hydroxycholesterol in the hippocampus, which suggests that pTau accumulation is associated with reduced cholesterol synthesis and cholesterol turnover in mutant mice. This is consistent with proteomic and transcriptomic signatures in *MAPT* P301S (35, 37) and *MAPT* P301S and G272V (36) mutant mice in previous studies. Cholesterol homeostasis is also greatly affected particularly in the hippocampus in tauopathies like AD (74). Decreased levels of lanosterol have also been reported in AD brain tissue, without changes in free cholesterol (25). Also, 24S-hydroxycholesterol, via increase in lanosterol and desmosterol, was reported to decrease atrophy and motor impairments in a model of Huntington’s disease (75).

It is striking that pTau accumulation associates with decreased cholesterol synthesis but increased cholesterol storage (CE). While CE only constitutes a small fraction of total cholesterol in the brain (76, 77), it serves as a marker for altered cholesterol metabolism. A possible explanation for the observed changes in cholesterol metabolism could be that animals with more advanced disease (more pTau build-up) have increased influx of lipids from the periphery into the brain through a compromised blood brain barrier (BBB) (78, 79), as BBB damage has been reported in *MAPT* P301S mice (80). Alternatively, increased entry of cholesterol into the brain might be a physiological process triggered by inflammation, as for example, microglia are known to uptake ApoA1 from the periphery (81), facilitating lipid delivery into the brain. Testing these hypotheses would require additional follow-up studies. In this respect, it is interesting to note that we observed diet-derived plant sterols to be increased in the brain of mutant mice with high pTau, possibly indicative of altered brain-peripheral lipid interactions (82, 83).

Although our findings provide novel insights into the role of Tau in lipid biology, we recognize the limitations of our study, which focuses on a single age group of female *MAPT* P301S mice. Investigating factors like age, sex, and brain regions in vivo, as well as different cell types in vitro, will reveal additional nuances in *MAPT* P301S-associated lipid signatures. Given that lipidome complexity increases from mice to humans (84) and *MAPT* P301S’s lipid effects may differ between species, these differences must also be taken into account. Further studies that track lipid changes at different stages of Tau pathology in larger cohorts are needed to delineate the temporal sequence of lipid alterations and their causal relation to Tau hyperphosphorylation and aggregation.

Overall, our data indicates that the FTLD-associated *MAPT* P301S mutation and pTau accumulation strongly and distinctly affect brain lipid metabolism in vivo.

## Data Availability

All data supporting the findings of this study are available within the paper and its Supplementary Information. Lipidomics data presented here will be added to the Neurolipid Atlas repository [https://neurolipidatlas.com/].

## Supplemental Data

This article contains supplemental data.

## Conflict of interest

T. V. and M. L. declare no conflict of interest; they are full-time employees of InnoSer (Synaptologics BV), a private company that provides in vivo CRO services. All other authors declare that they have no known competing financial interests or personal relationships that could have appeared to influence the work reported in this paper.
