# Supplementary material for: Characterization of the brain lipidome associated with frontotemporal lobar degeneration MAPT P301S mutation
Source: J Lipid Res. 2025 Nov 27;67(1):100952. doi: 10.1016/j.jlr.2025.100952 (PMC12796110; doi:10.1016/j.jlr.2025.100952)

1.
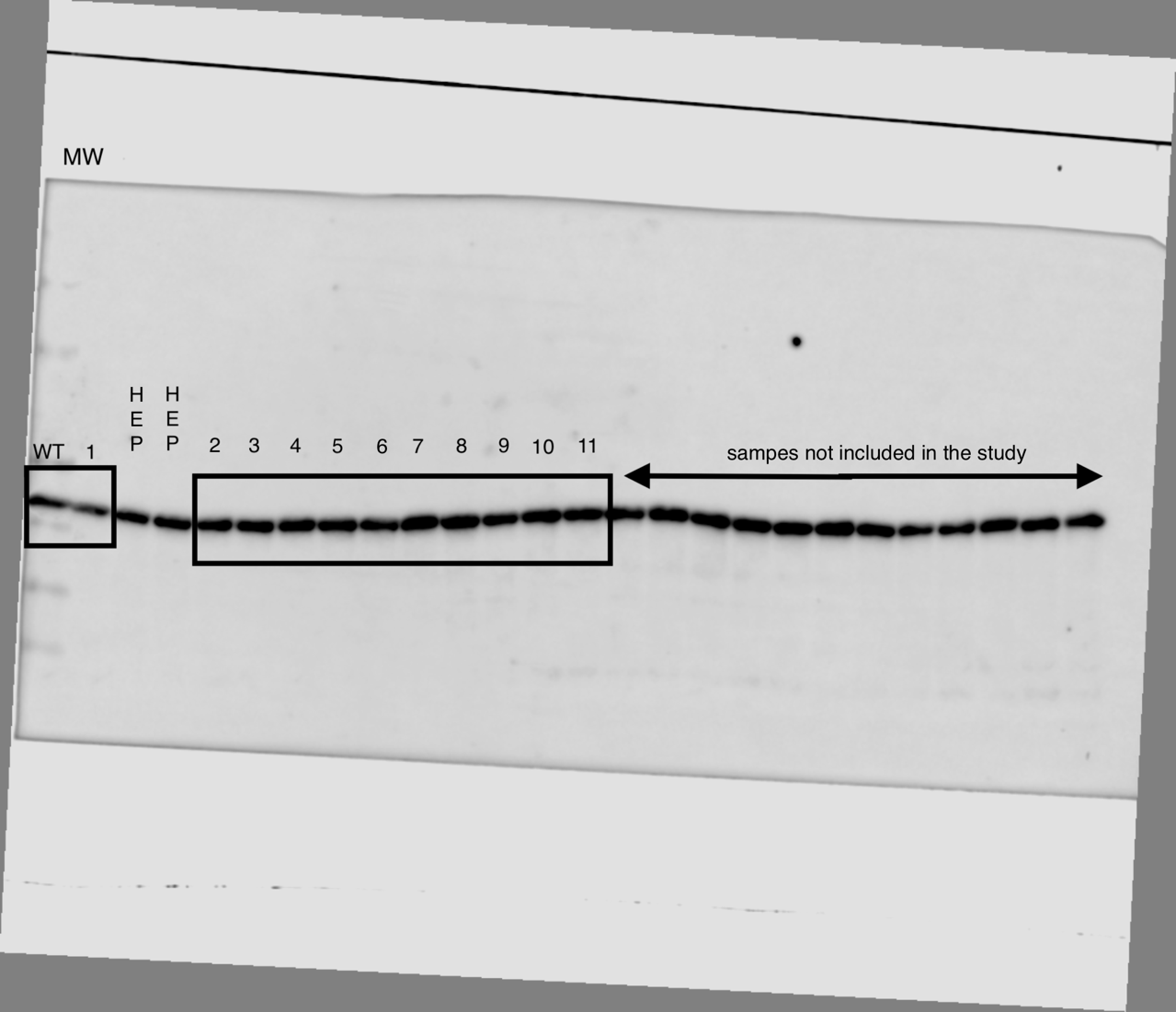
Uncropped blot from hippocampal samples staining β-actin (from AT8-stained blot).
2.
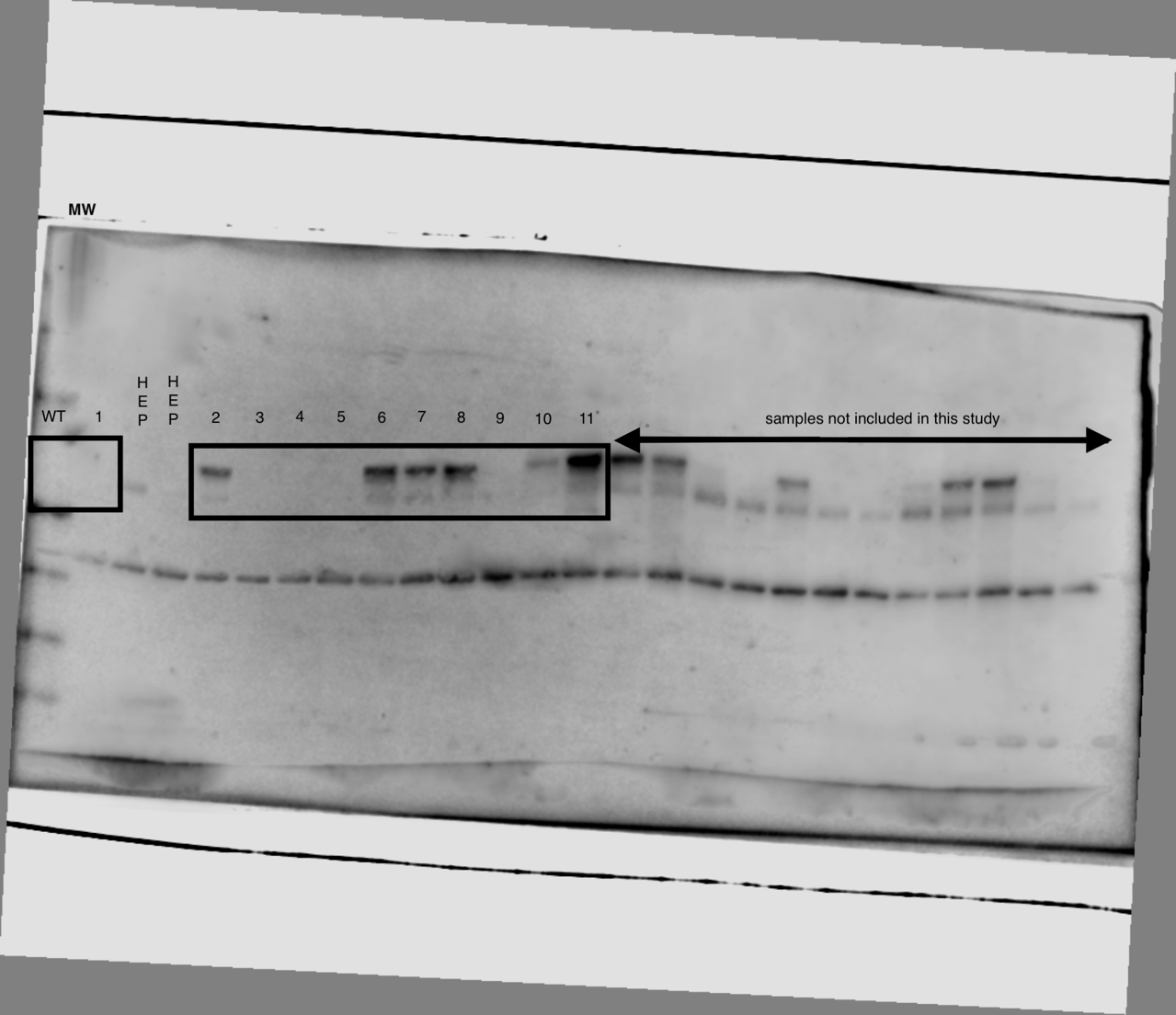
Uncropped blot from hippocampal samples staining AT8.
3.
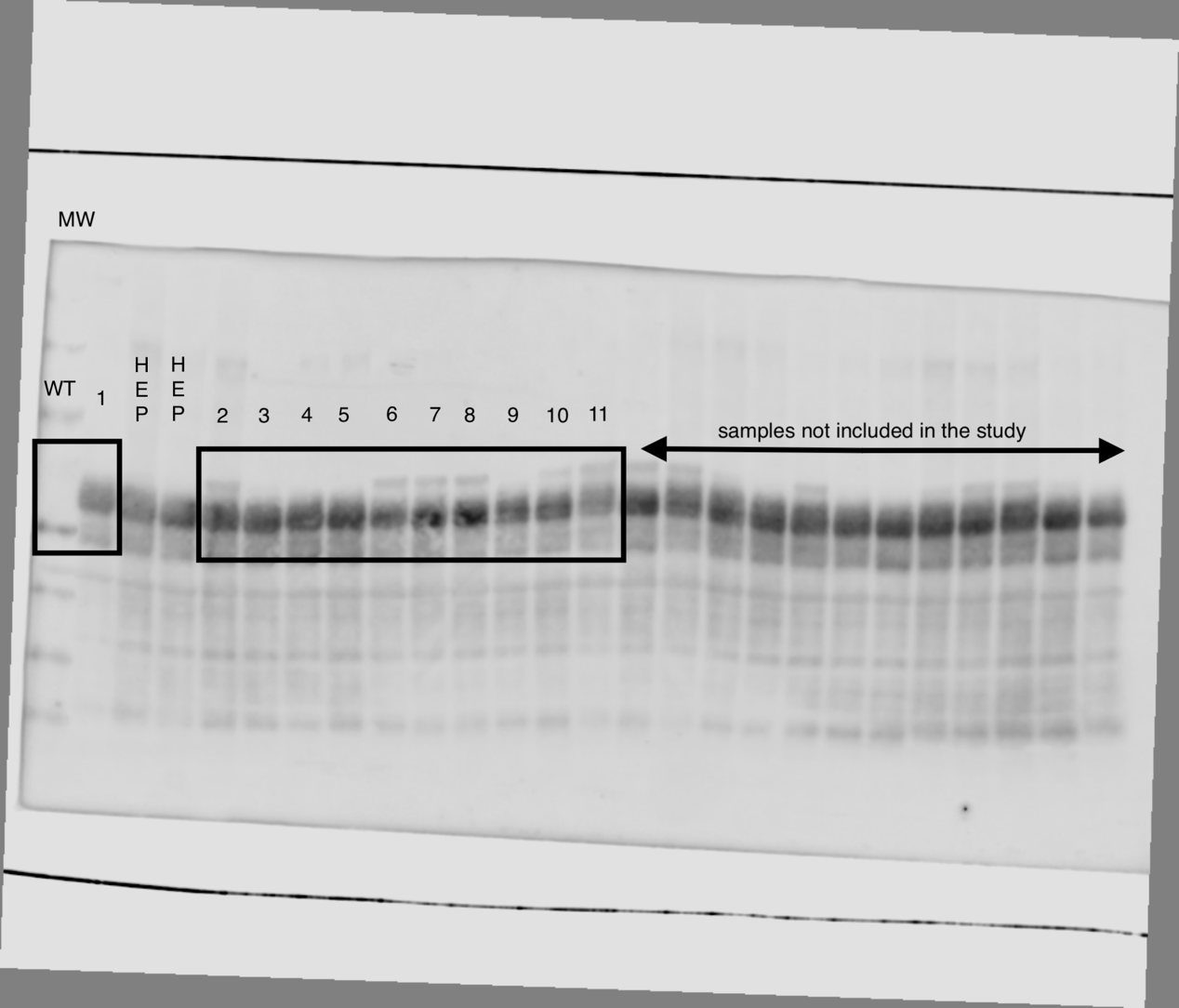
Uncropped blot from hippocampal samples staining total Tau (same membrane as AT8). Above: image corresponding to low contrast. Below: image corresponding to increased contrast.


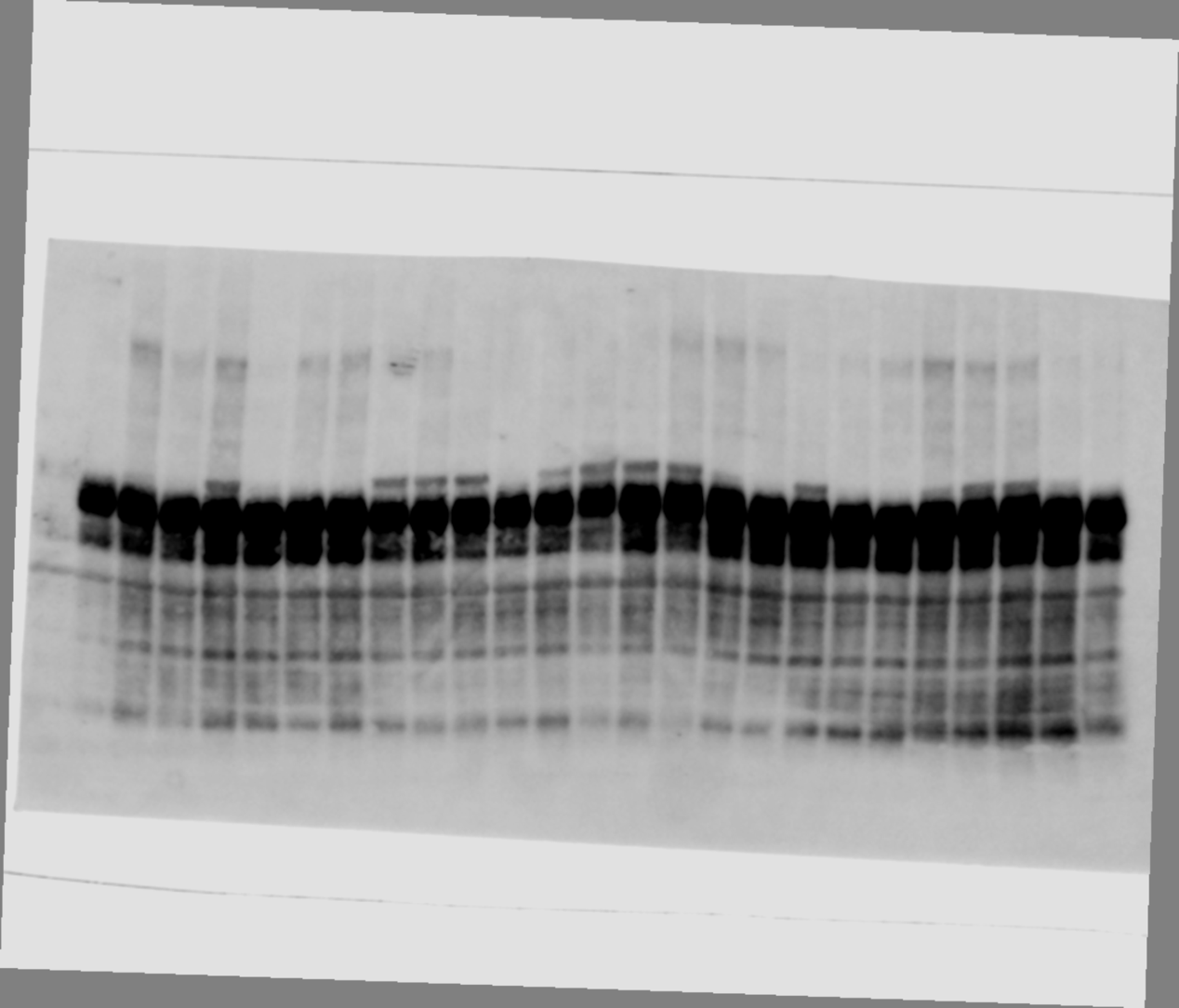


1.
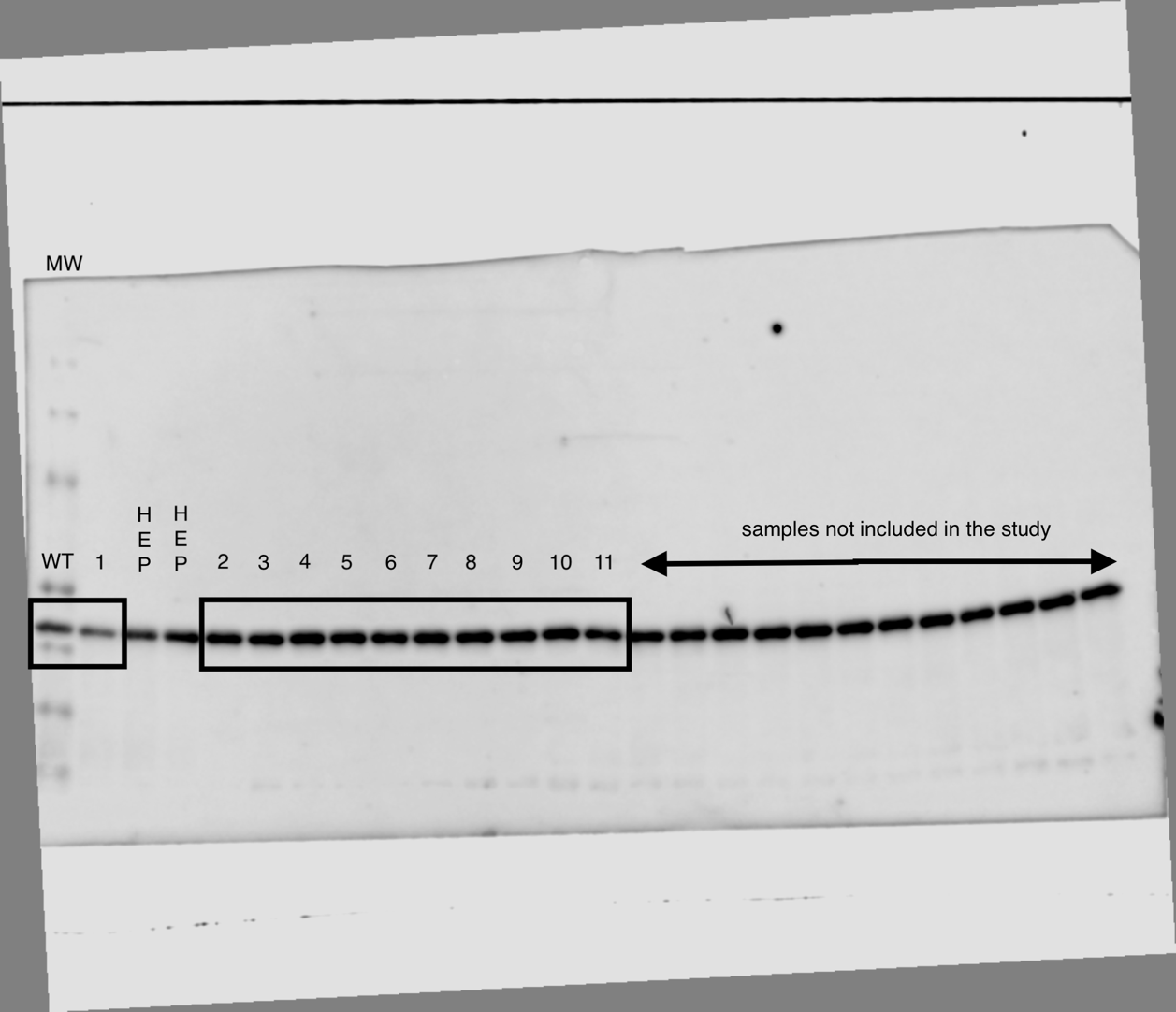
Uncropped blot from hippocampal samples staining β-actin (from PHF1-stained blot).
2.
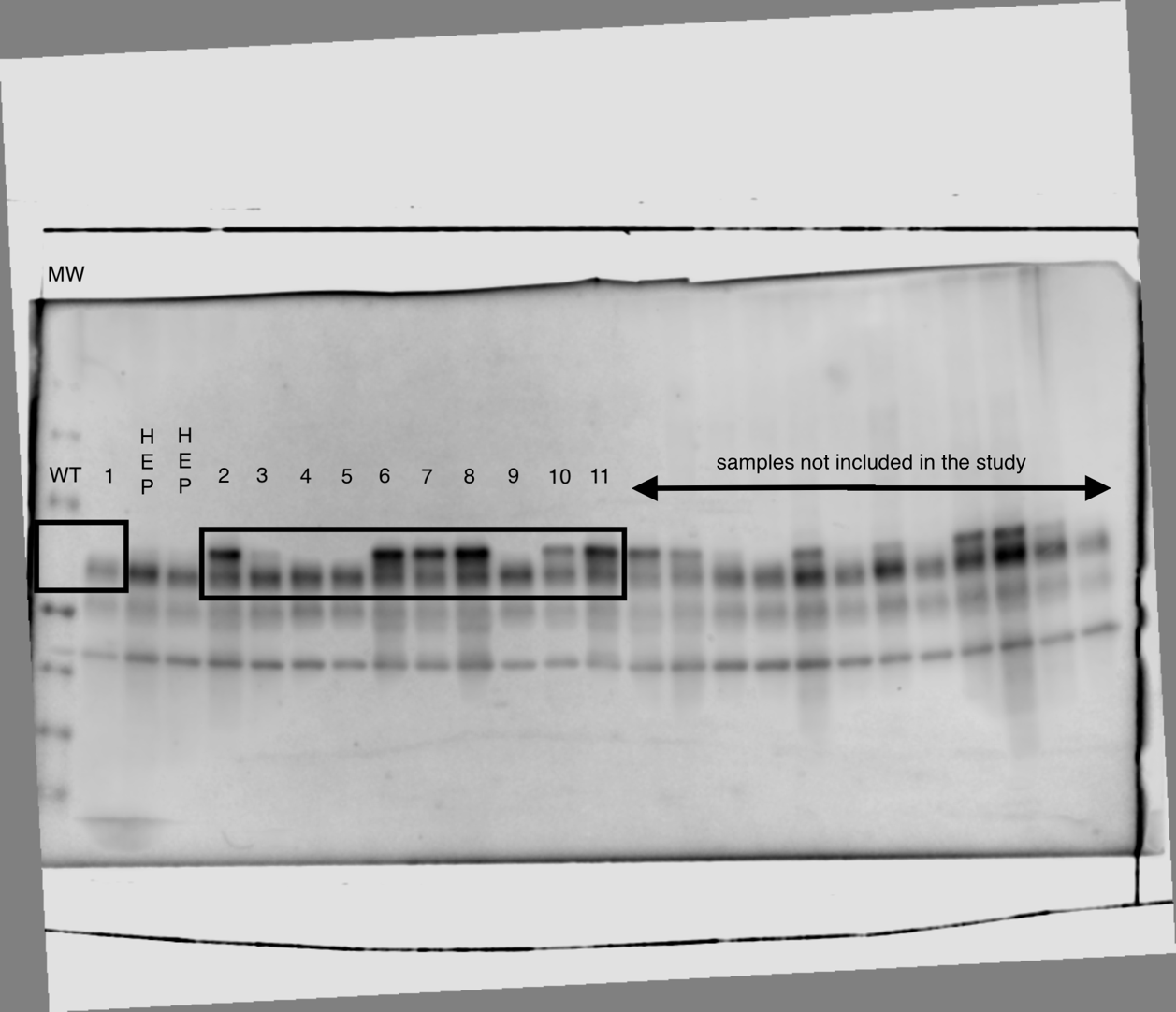
Uncropped blot from hippocampal samples staining PHF1.
3. Uncropped blot from cortical samples staining β-actin (from AT8-stained blot).


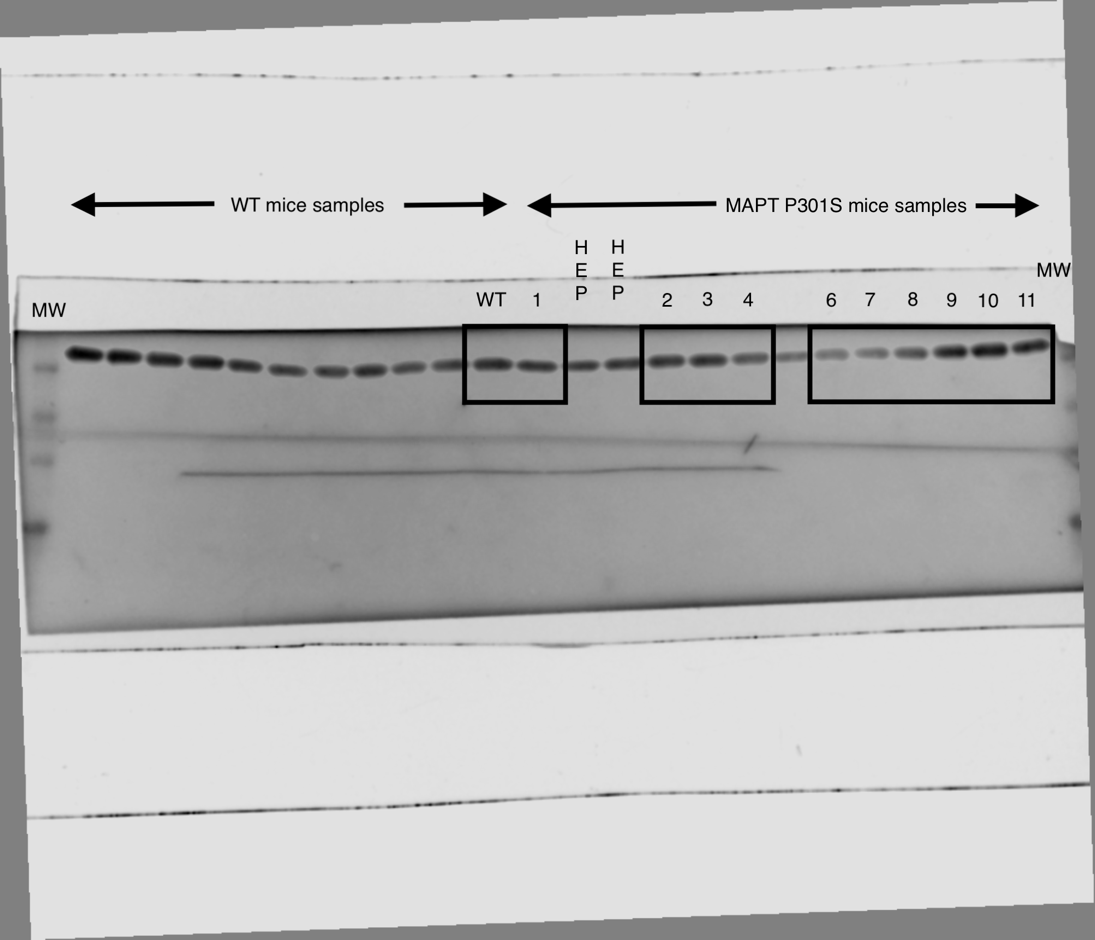


1. Uncropped blot from cortical samples staining AT8.


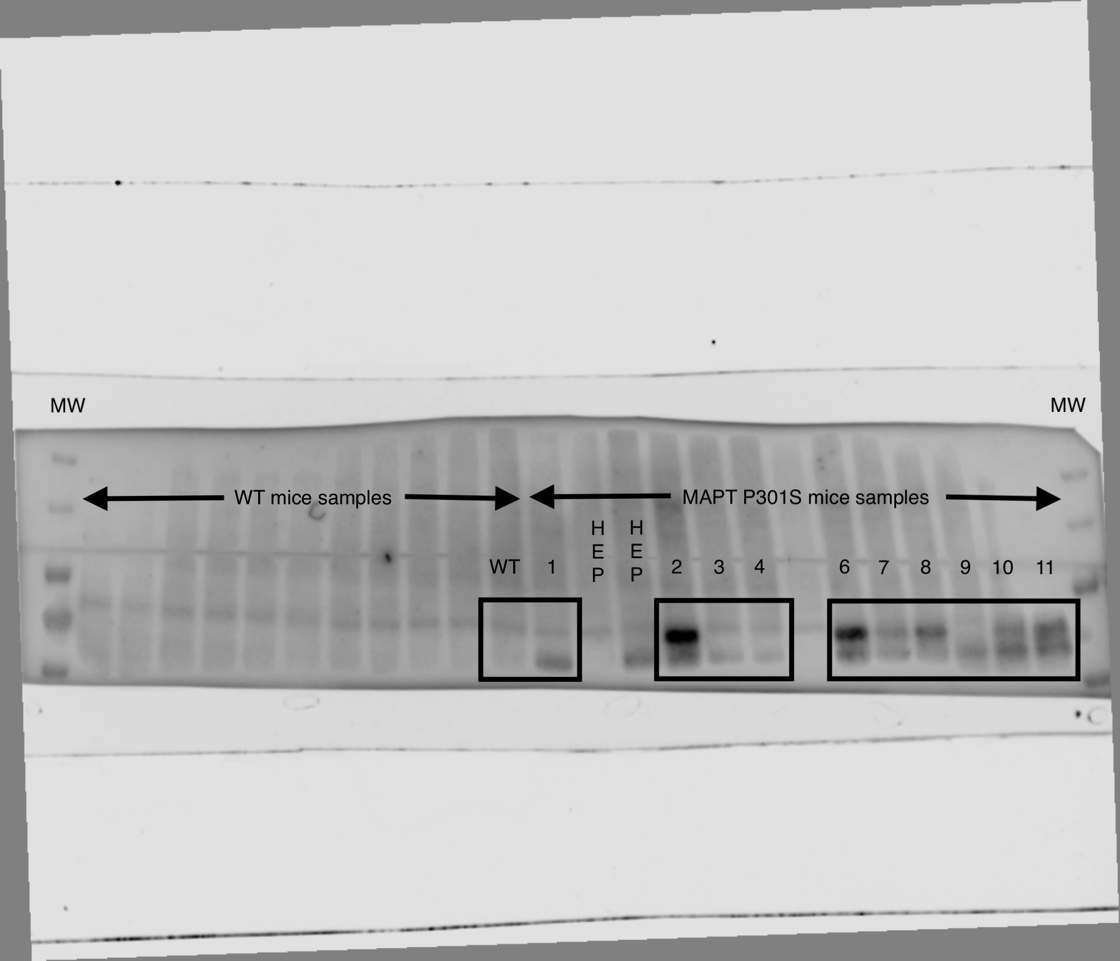


1. Uncropped blot from cortical samples staining total Tau (same membrane as AT8). Above: image corresponding to low contrast. Below: image corresponding to increased contrast for visualization of signal in WT samples.


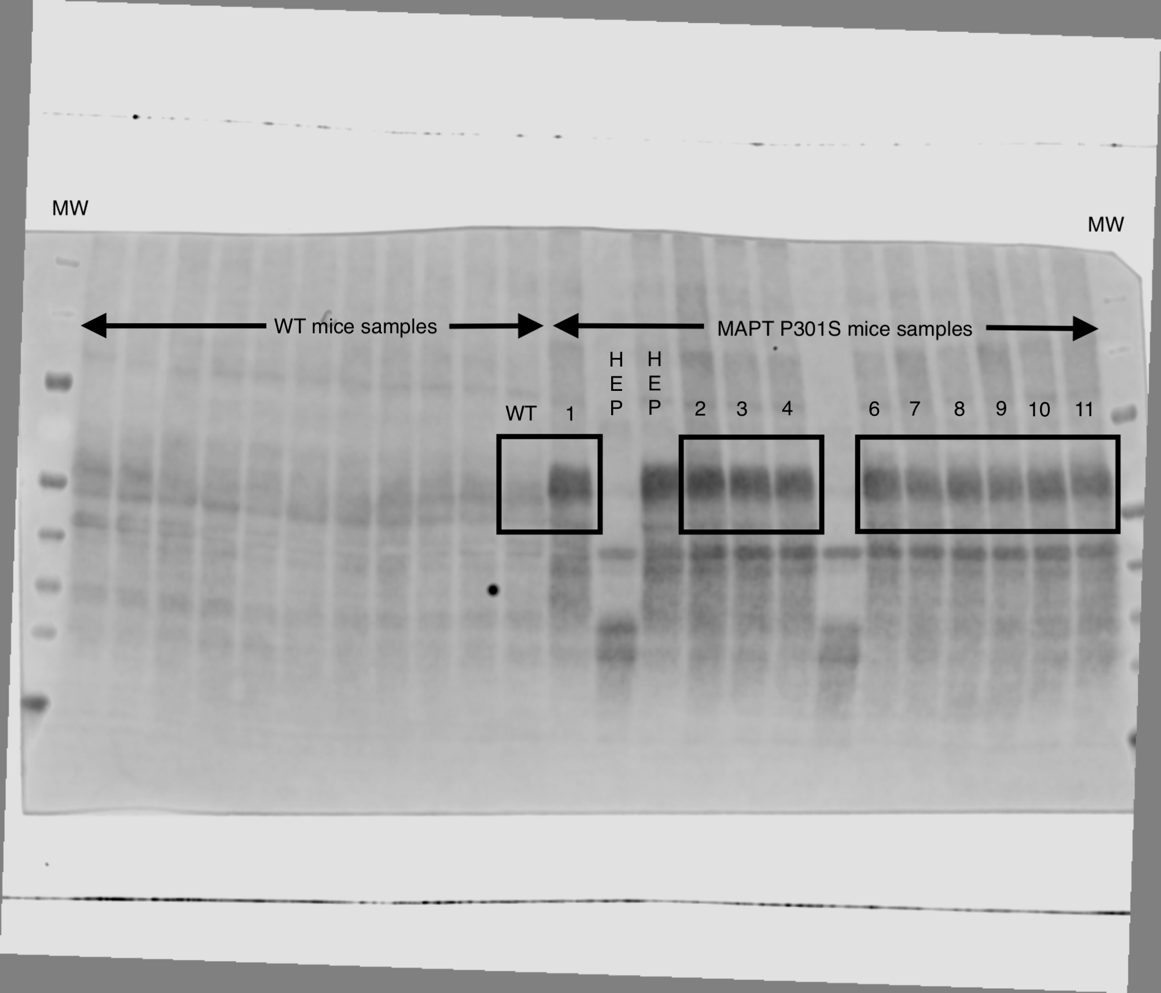

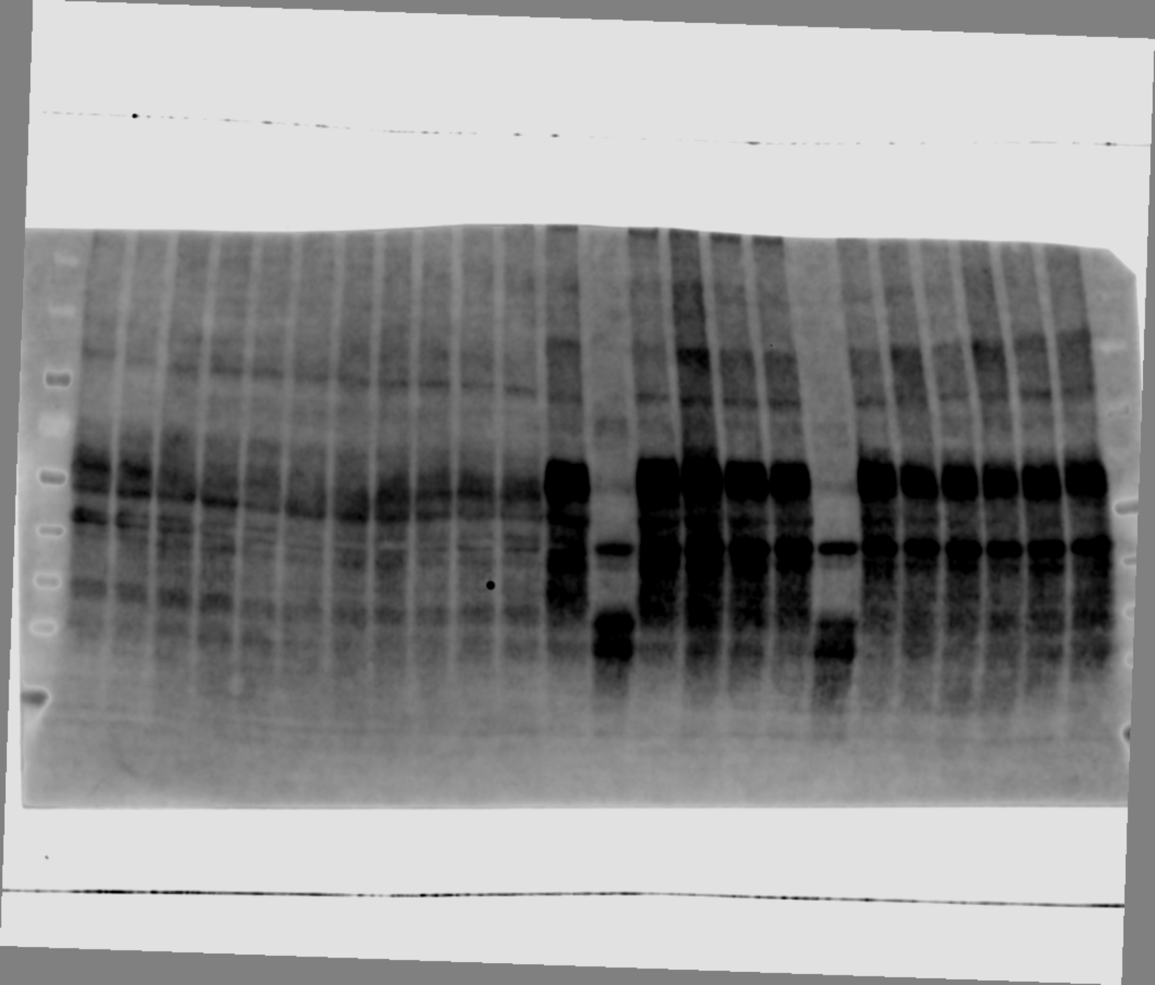


1.
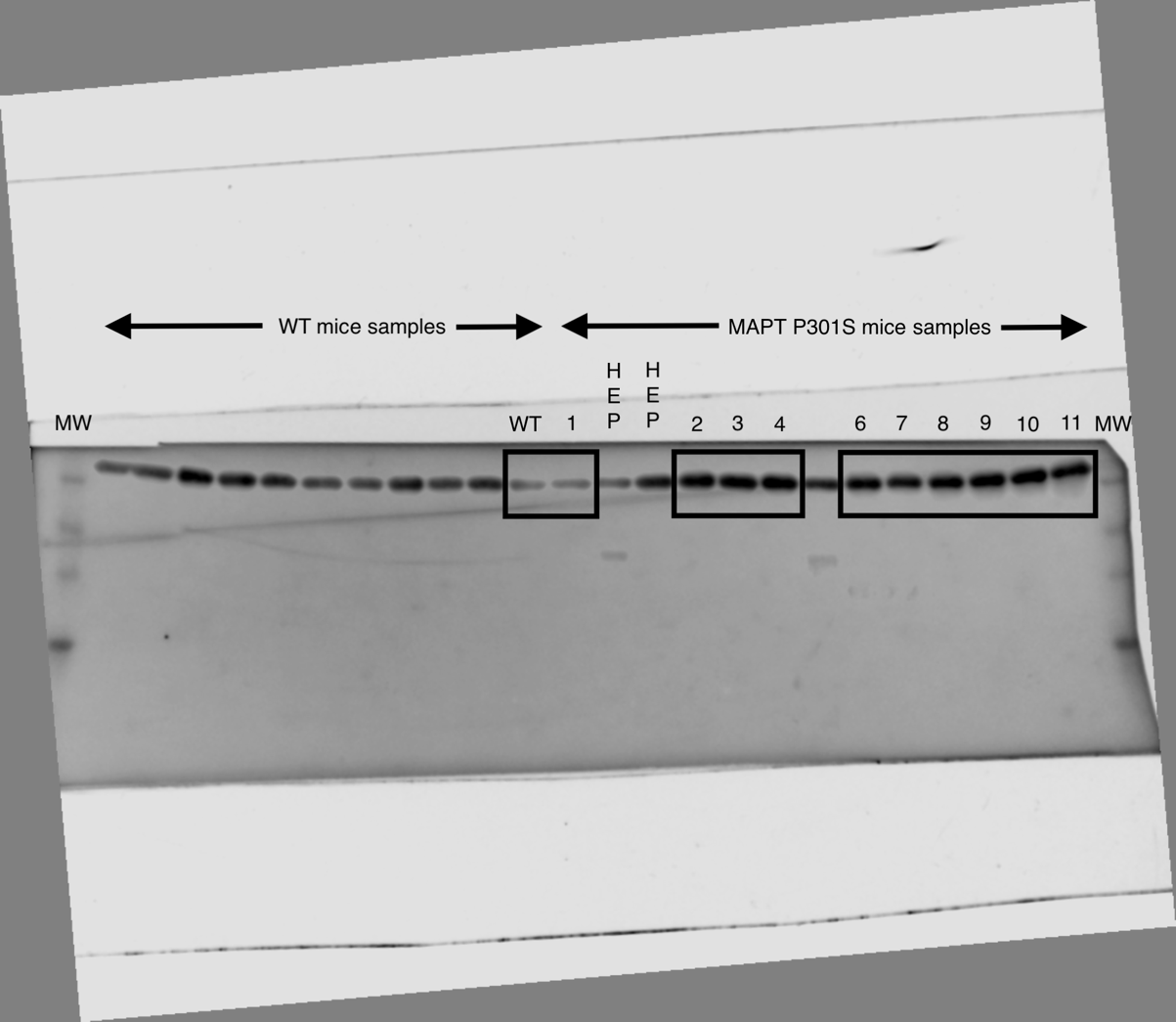
Uncropped blot from cortical samples staining β-actin (from PHF1-stained blot).
2. Uncropped blot from cortical samples staining PHF1


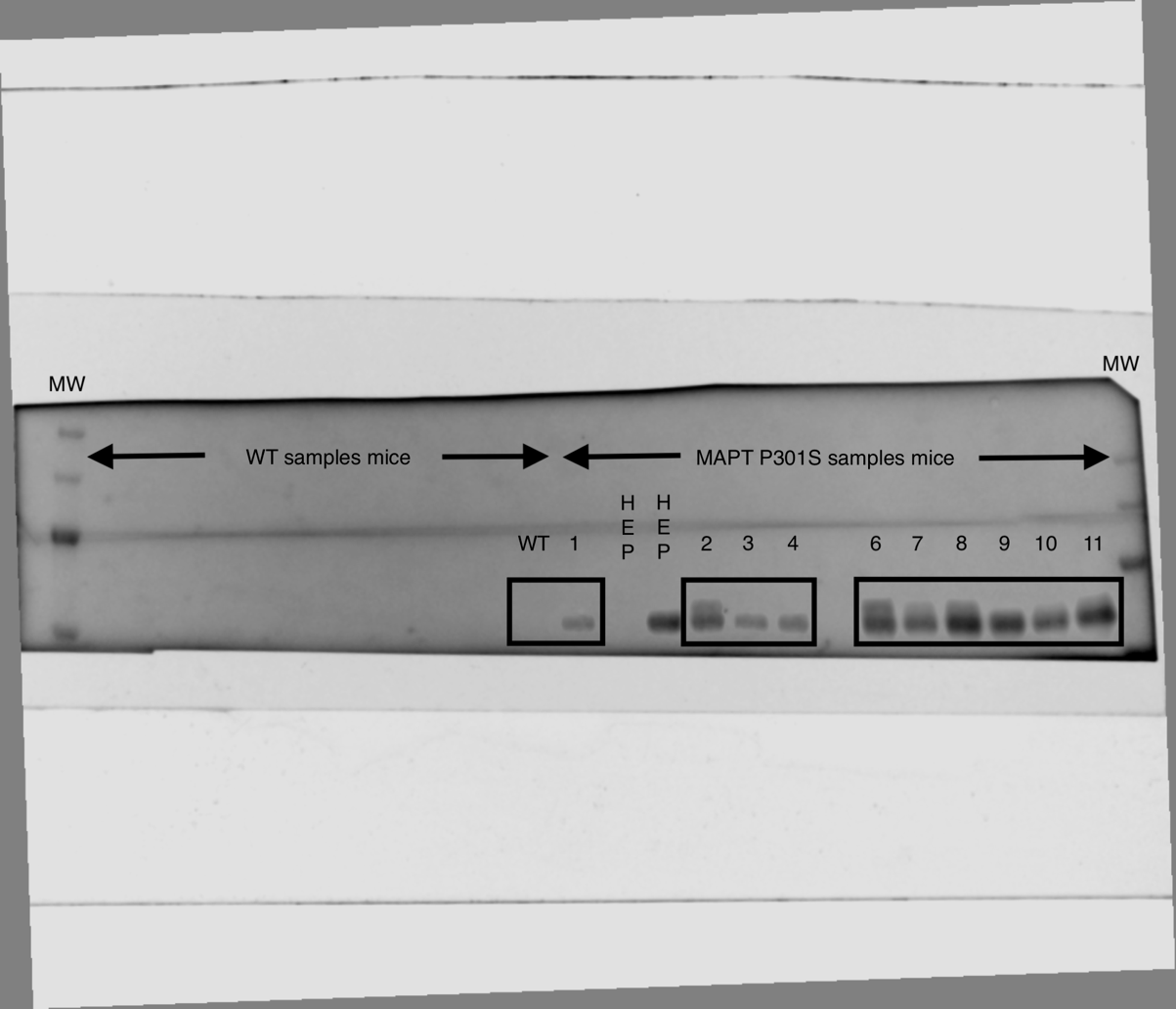

Supplement: Supplementary Data 2 [file mmc2.docx]
