# Supplementary material for: Characterization of the brain lipidome associated with frontotemporal lobar degeneration MAPT P301S mutation
Source: J Lipid Res. 2025 Nov 27;67(1):100952. doi: 10.1016/j.jlr.2025.100952 (PMC12796110; doi:10.1016/j.jlr.2025.100952)
Supplement: Supplementary Figures [file mmc3.docx]

**SUPPLEMENTARY FIGURES**

**
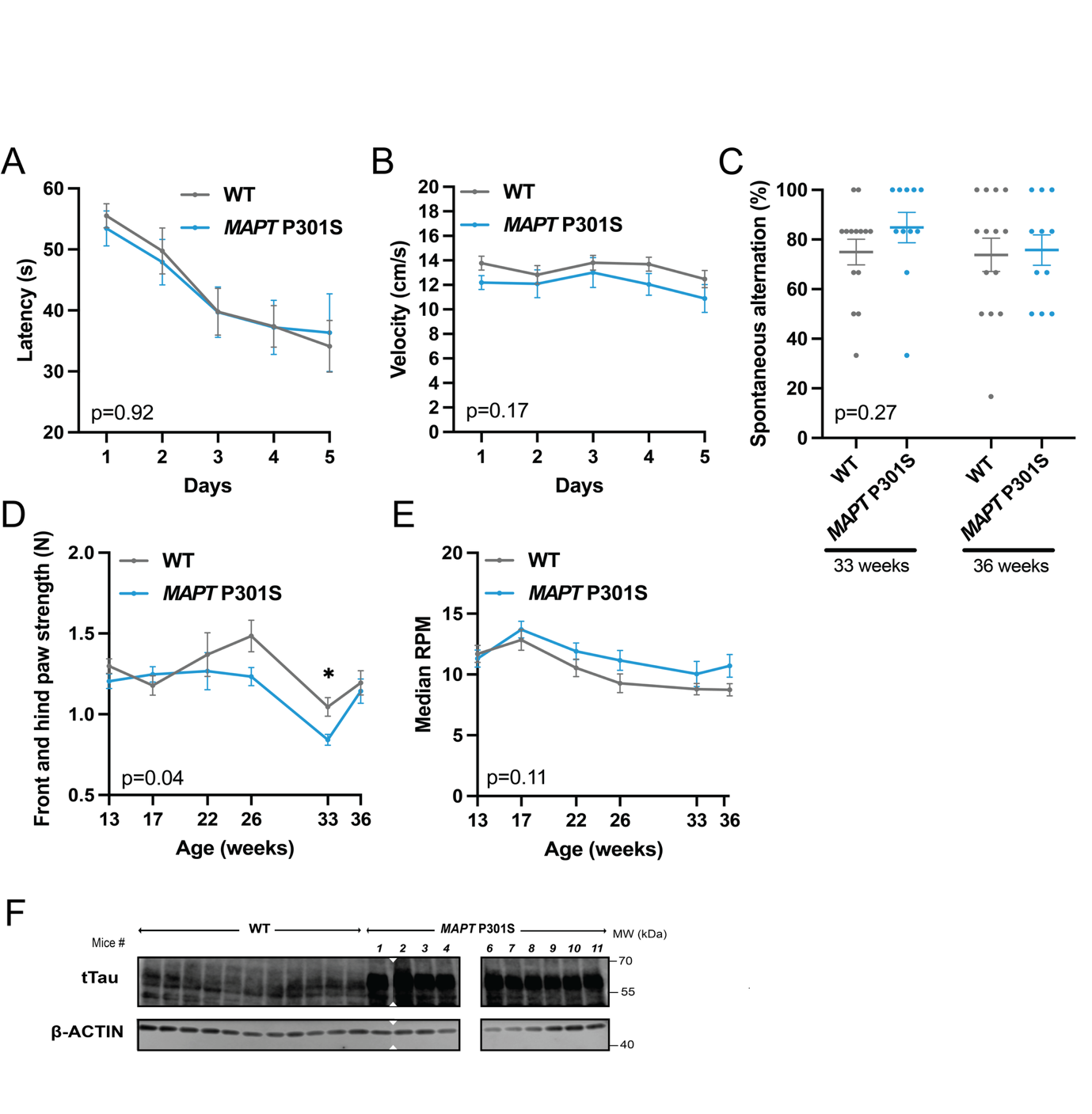
**

***Figure S1****.* ***Study of behavior in MAPT P301S female mice****.*

***A,B)*** *Morris Water Maze test at 38 weeks assessing latency to platform (A) and swimming velocity (B) in WT and MAPT P301S mice shows no significant differences between genotypes (latency: F (1, 23) = 0.01; p = 0.92. Swimming velocity: F (1, 23) = 1.97; p = 0.17) or the interaction between genotype and time (latency: F (4, 92) = 0.15; p = 0.96. Swimming velocity: F (4, 92) = 0.33; p = 0.86).*

***C)*** *Spontaneous alternation between right and left arms at 33 or 36 weeks of age in WT and MAPT P301S mice during T-maze showing no significant differences between genotypes (F (1, 23) = 1.28; p = 0.27) or the interaction between genotype and time (F (1, 23) = 0.33; p = 0.57).*

***D)*** *Grip strength for front and hind paws calculated longitudinally from 13 to 36 weeks in WT and MAPT P301S mice shows significantly worse performance of MAPT P301S mice when comparing genotypes (F (1, 23) = 4.80; p = 0.04), but the interaction between genotype and time was not significant (F (5, 115) = 1.04; p = 0.40). Post-hoc analysis revealed genotype differences are significant only at week 33.*

***E)*** *Rotarod data from 13 to 36 weeks presented as median RPM in WT and MAPT P301S mice shows no significant differences between genotypes (F (1, 23) = 2.71; p = 0.11) or the interaction between genotype and time (F (5, 115) = 0.98; p = 0.43). WT mice show a trend towards worse performance compared to the MAPT P301S mice in the rotarod, potentially due to their higher body weight.*

***F)*** *Western blot images of total Tau (tTau) and beta actin (β-actin) levels in lysates from cortex of WT mice (n=11) and MAPT P301S (n=10) mice at 9 months of age.* *Contrast was increased for visualization of tTau signal in WT mice.* *Sections where the blots were cut are indicated with white triangles.*

*Data are presented as mean ± SEM. Two-way ANOVA was applied for all behavioral tests, followed by multiple comparison testing with Bonferroni correction. Probability level was set at 95%. p-values for genotype comparisons are shown in the graphs. If applicable, p-values from post-hoc analysis are also displayed, *=p<0.05. WT mice are depicted in grey (n=14), MAPT P301S mice are depicted in blue (n=11).*

*
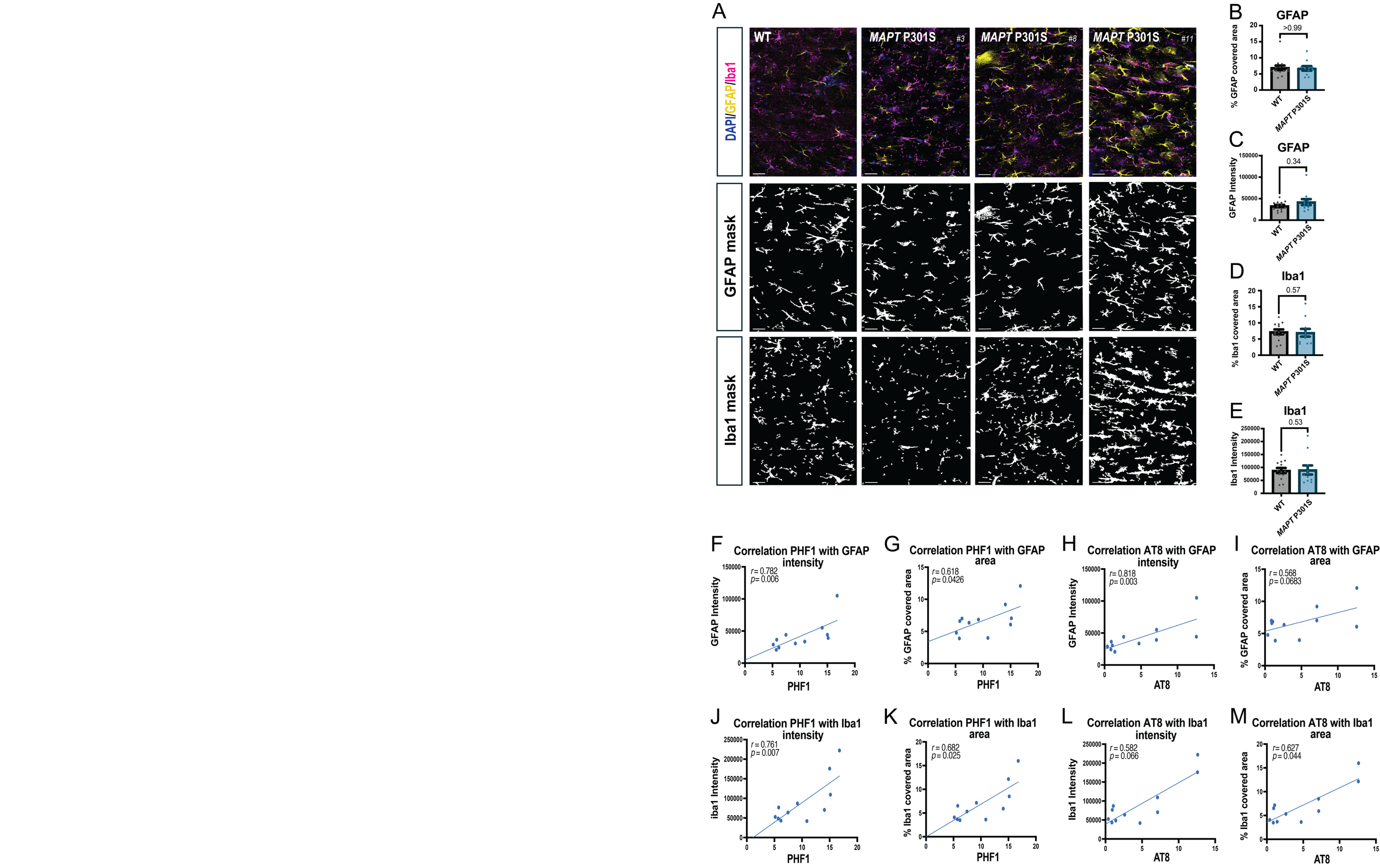
*

***Figure S2. MAPT P301S mice with high pTau present astro- and microgliosis in hippocampus.***

***A-E)*** *Representative immunofluorescence images with corresponding masks (A) and quantification (B-E) of GFAP-covered area (B) and intensity (C) and Iba1-covered area (D) and intensity (E) in GFAP- and Iba1-stained CA1 region of hippocampus (HC) of wild-type (WT) and MAPT P301S mice. Mice genotype and mice identifier number are indicated. Yellow=GFAP, Magenta=Iba1, Blue=DAPI. Scale bars: 20µm. Each data point represents one individual mouse; WT mice are depicted in grey (n=14), MAPT P301S mice are depicted in blue (n=11).*

***F-I)*** *Correlation analysis of relative abundance of pTau PHF1 (F,G) and AT8 (H,I), as measured by western blot, with GFAP intensity (F, H) and area (G, I) in hippocampus as measured by immunofluorescence. Spearman correlation coefficient (r) and p value are indicated. n=11 MAPT P301S mice.*

***J-M)*** *Correlation analysis of relative abundance of pTau PHF1 (J,K) and AT8 (L,M), as measured by western blot, with Iba1 intensity (J, L) and area (K, M) in hippocampus as measured by immunofluorescence. Spearman correlation coefficient (r) and p value are indicated. n=11 MAPT P301S mice.*

*
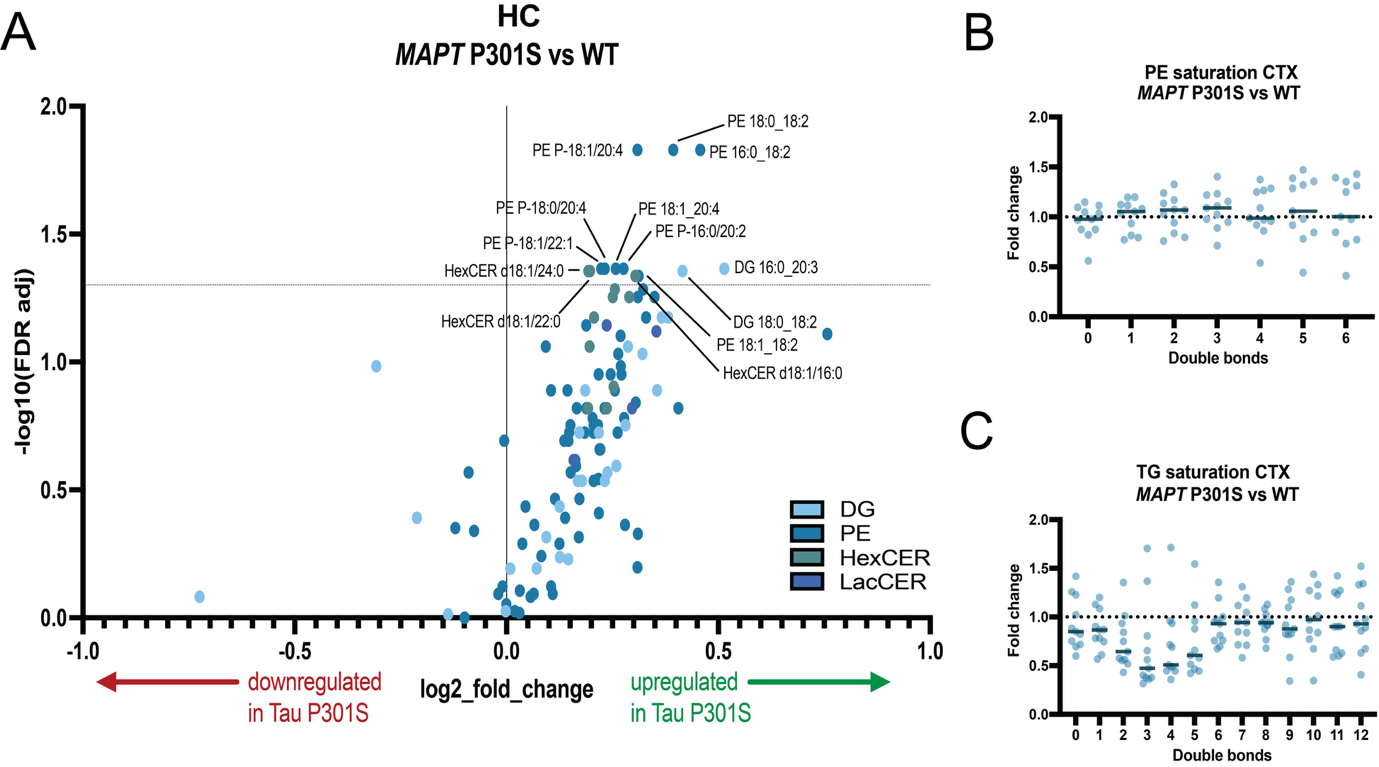
*

***Figure S3. Brain lipidomics analysis of MAPT P301S.***

***A)*** *Volcano plot of altered lipid species from increased lipid classes lactosylceramide (LacCER), hexosylceramide (HexCER), diglyceride (DG) and phosphatidylethanolamine (PEs) in hippocampus (HC) of MAPT P301S vs wild-type (WT) mice. Significantly increased species are labeled.*

***B,C)*** *Fold change in PEs (B) and triglycerides (TGs) (C) with indicated number of double bonds (unsaturation) in cortex of MAPT P301S vs WT mice.* *Each data point represents one individual MAPT P301S mouse (n=11).*

*Mann-Whitney U test with Benjamini-Hochberg correction for multiple comparisons was applied with a probability level set at 95%.*


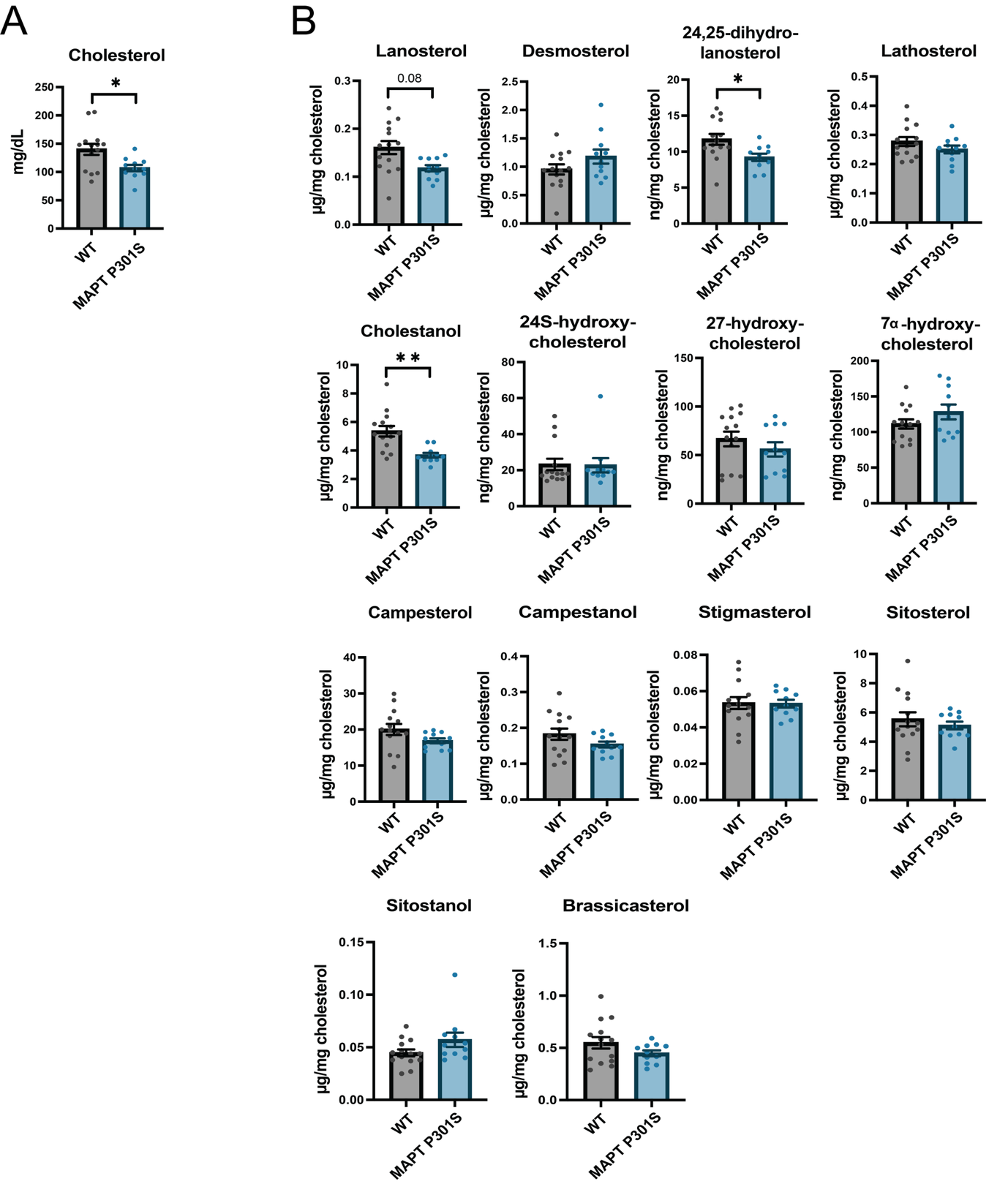


***Figure S4.*** ***Analysis of sterols, oxysterols and phytosterols in plasma of*** ***MAPT P301S mice.***

***A)*** *Analysis of total cholesterol in plasma of MAPT P301S and wild-type (WT) mice.*

***B)*** *Analysis of sterols, oxysterols and phytosterols normalized to total cholesterol in plasma of MAPT P301S and WT mice.*

*Error bars represent mean ± SEM. Each data point represents one individual mouse. Mann-Whitney U test with Benjamini-Hochberg correction for multiple comparisons was applied with a probability level set at 95%. *=p<0.05, **=p<0.01. WT mice are depicted in grey (n=14), MAPT P301S mice are depicted in blue (n=11).*


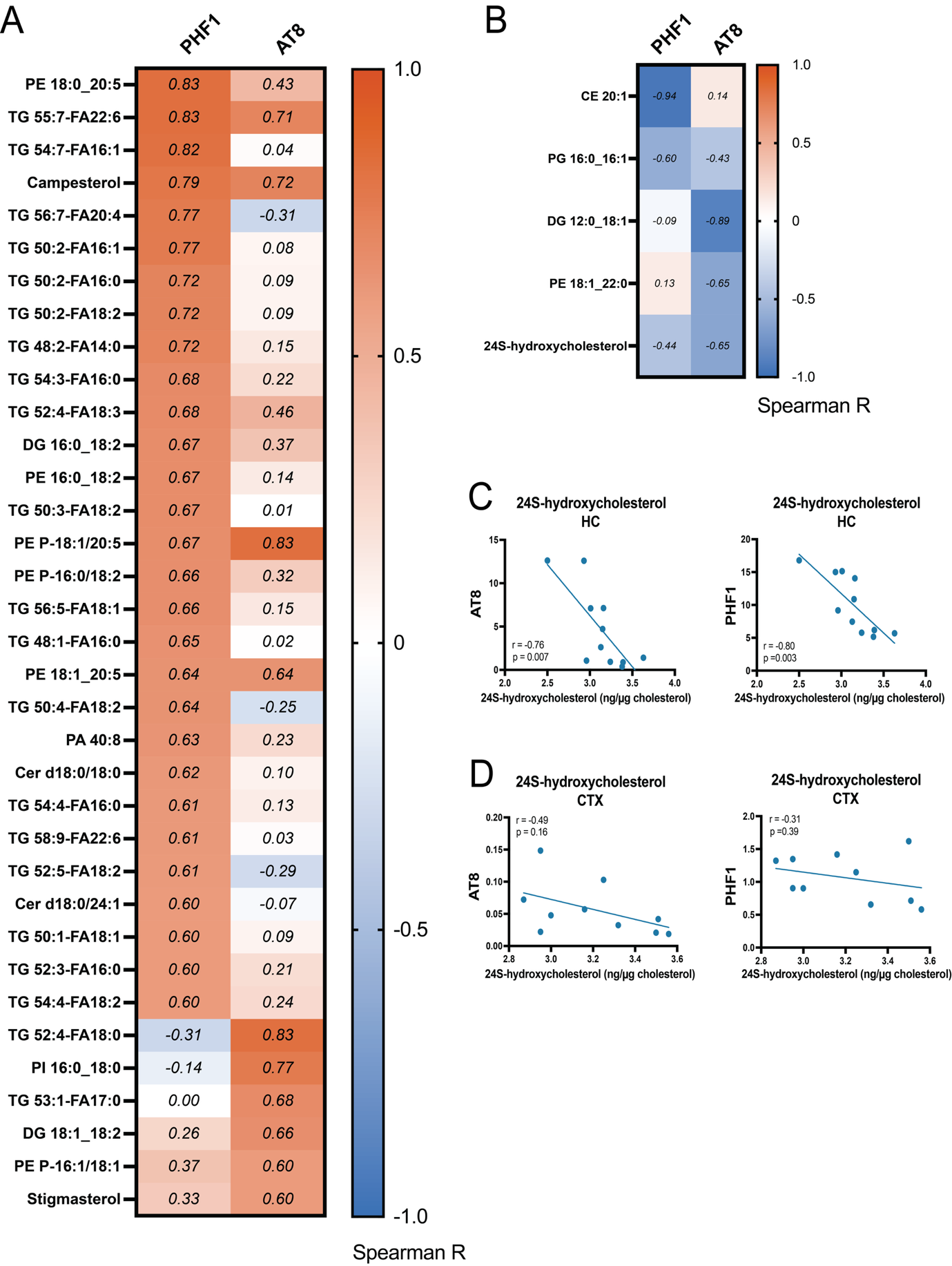


***Figure S5. Correlation of lipid profile and 24S-hydroxycholesterol with pTau in the brain of MAPT*** ***P301S mice.***

***A,B)*** *Heatmap showing Spearman correlation coefficient (r) of the 40 highest positively correlating (A) and the 10 highest negatively correlating (B) lipid species (from lipidomics and sterol panel) with relative abundance of pTau AT8 and PHF1 epitopes in cortex of MAPT P301S mice (n=10).*

***C, D)*** *Linear* *correlations between 24S-hydroxycholesterol and relative abundance of pTau epitopes AT8 and PHF1 as determined by western blot in hippocampus (HC, C) and cortex (CTX, D) of MAPT P301S mice. Each data point represents one individual mouse. Pearson’s correlation coefficient (r) is shown for each comparison and p value is presented with a probability level set at 95%. n=11 HC MAPT P301S, n=10 CTX MAPT P301S.*


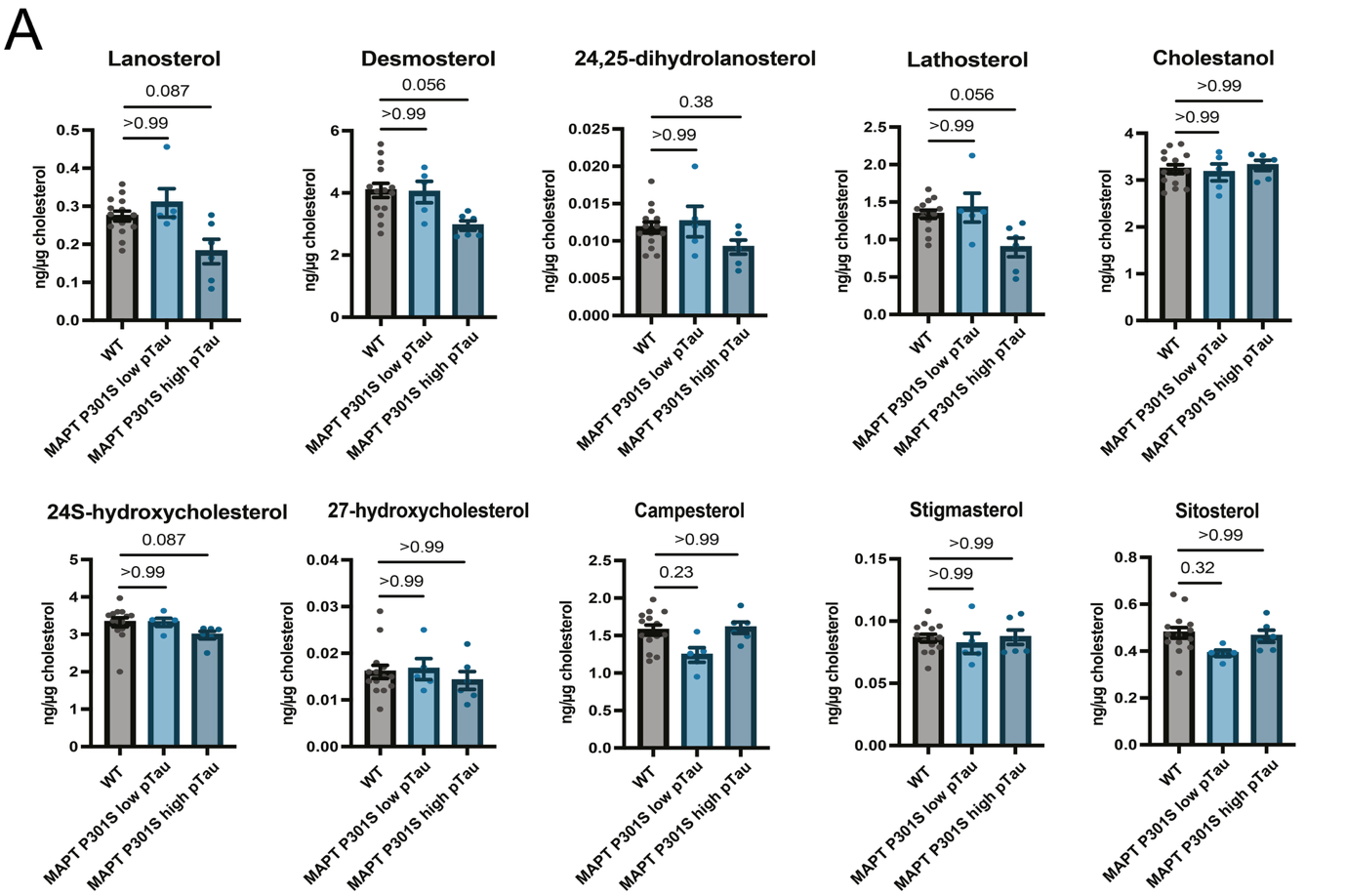


***Figure S6. Hippocampal sterols and triglyceride species in WT mice and MAPT P301S mice with low and high pTau.***

***A)*** *Analysis of sterols, oxysterols and phytosterols normalized to total cholesterol in hippocampus of MAPT P301S (stratified by low and high pTau levels) and WT mice. Error bars represent mean ± SEM. Each data point represents one individual mouse. Ordinary one-way ANOVA with Bonferroni correction was applied for normally distributed species, whereas Krustall-Wallis test with Dunn’s correction was used for non-normally distributed species, with a probability level set at 95%; followed by FDR correction for multiple comparisons. WT mice are depicted in grey (n=14), MAPT P301S mice with low pTau are depicted in light blue (n=5), MAPT P301S mice with high pTau are depicted in dark blue (n=6).*
